# A systematic review exploring perceptions of Tourette syndrome and tic disorders using the common-sense model of illness representations

**DOI:** 10.1080/08870446.2025.2502515

**Published:** 2025-05-14

**Authors:** Charlotte Petter, Kareem Khan, Camilla Babbage, E. Bethan Davies

**Affiliations:** a School of Medicine, University of Nottingham, Nottingham, UK; b Mental Health & Clinical Neurosciences, NIHR Nottingham Biomedical Research Centre, School of Medicine, NIHR MindTech Medtech Cooperative, University of Nottingham, Nottingham, UK; c MindTech HealthTech Research Centre, Institute of Mental Health, School of Medicine, The University of Nottingham, Nottingham, UK; d School of Applied Social Sciences, De Montfort University, Leicester, UK

**Keywords:** Tourette syndrome, tic disorders, systematic review, illness perceptions, illness beliefs

## Abstract

**Objective:**

Tic disorders (TDs) are neurodevelopmental conditions characterised by tics and typically appear during childhood. The Common Sense Model of Self-Regulation (CSM) provides a useful theoretical framework for understanding health beliefs in people with TDs – and parents’ beliefs given ‘shared’ illness experiences between child and parent. Exploring health beliefs in adults can also provide insight as to how TD-related beliefs may evolve over time. This systematic review aimed to use the CSM to synthesise findings from published studies exploring illness perceptions in people with TDs and parents.

**Methods:**

Six databases were searched for studies reporting findings assessing perceptions and beliefs of TDs that aligned with ≥1 CSM illness representation dimension. Forty-four studies were eligible and narratively synthesised.

**Results:**

The evidence particularly highlights the negative *consequences* of TDs upon employment opportunities, schooling and education, social lives and relationships – with experiences of stigma and discrimination weaving throughout these consequences. Findings from several studies reflecting *emotional responses* report feelings of self-consciousness, abnormality, and anxiety arising from TDs.

**Conclusion:**

Findings have identified potential implications for research and practice, including identifying TD-related knowledge and beliefs that could be addressed through psychoeducation, and physiological and psychological outcomes which could be addressed through appropriate interventions.

**Protocol registration:**

PROSPERO CRD42023446800

## Introduction

**Figure 1.**
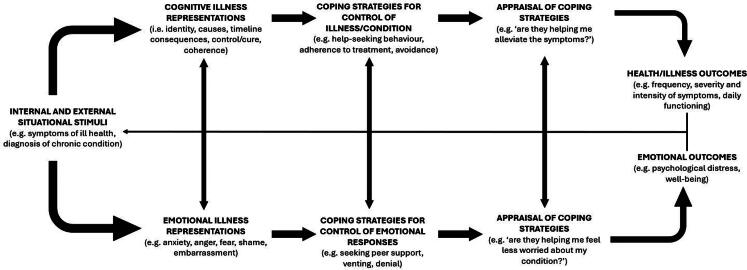
The Common Sense Model of Self-Regulation (CSM) (1, 3, 101) (Leventhal et al., 1980).

When faced with a health threat - such as experiencing new symptoms or being diagnosed with a chronic condition - an individual must make sense of this information, and in doing so they form their own beliefs and attitudes that guide their behaviour and management of the health threat (Baines & Wittkowski, 2013; Leventhal & Brissette, 2012). One model theorising how individuals respond to health threats is the Common-Sense Model of Self-Regulation (CSM; also called the Self-Regulation model) (Leventhal & Brissette, 2012). This socio-cognitive model theorises two pathways reflecting a person’s cognitive and emotional response to the presence of a health threat. The pathways work in parallel to form illness beliefs, which in turn guide the individual’s use of coping strategies to manage their cognitive and emotional responses (Figure 1) (Baines & Wittkowski, 2013; Bear et al., 2021). The CSM comprises five dimensions contributing to a person’s cognitive illness representation—the beliefs a person holds about health and illness—(Leventhal & Brissette, 2012); *Identity*, the identification and labelling of symptoms/illness; (Baines & Wittkowski, 2013); *Cause*, the perceived cause of the illness; (Bear et al., 2021); *Timelin*e, the perceived length and course of the illness; (Moss-Morris et al., 2002); *Consequences*, the beliefs regarding the short and long-term impact of illness on an individual’s life; and (Hagger & Orbell, 2003); and c*ontrol/curability*, the beliefs regarding controllability and curability of the illness by the individual (*personal control*) and by treatment (*treatment control*) (Leventhal & Brissette, 2012; Moss-Morris et al., 2002). A sixth dimension—*illness coherence*—has also been proposed, reflecting whether the illness makes sense to the individual (Moss-Morris et al., 2002). The emotional pathway reflects the individual’s emotional response towards symptoms and illness (e.g. fear, anger, worry), which are defined as *emotional representations* (Bear et al., 2021). These illness representations are processed alongside each other to guide cognitive (e.g. information seeking, going through with treatment) and emotional (e.g. venting, relaxation techniques) coping strategies, which in turn can impact on illness and emotional outcomes (e.g. disease state, psychological well-being). According to the CSM, the individual appraises their coping strategies upon such outcomes and adjusts as needed (Baines & Wittkowski, 2013; Bear et al., 2021).

Illness representations are important for several reasons. Firstly, specific illness cognitions are linked with illness outcomes. In a meta-analysis of 45 studies that measured illness representations in a variety of chronic and short-term conditions (e.g. diabetes, myocardial infarction), Hagger and Orbell (Hagger & Orbell, 2003) found that beliefs relating to *consequences* and *illness identity* have been found to be significantly negatively associated with physical functioning, and furthermore beliefs within the *control/cure* dimension was found to be significantly negatively associated with psychological distress and disease state (Hagger & Orbell, 2003). Secondly, as proposed in the model, cognitive and emotional illness representations guide a person’s coping strategies, and both can impact upon illness outcomes (Hagger & Orbell, 2003). In a systematic review applying the CSM to inflammatory bowel disease, illness representations were found to either directly or indirectly positively mediate the relationship between disease outcomes, psychological distress and quality of life (Hayes et al., 2020). This can also expand onto coping styles—the behavioural and cognitive responses to manage experiencing illness - with two studies in the same review finding that emotional venting and disengagement were significantly associated with higher levels of anxiety and depression (Hayes et al., 2020). Thirdly, illness perceptions are linked to beliefs about treatment—which in-turn, could impact upon treatment adherence and clinical outcomes of illness. For example, parents of autistic children who attributed the cause of their child’s condition to external factors and being hereditary were more likely to try metabolic treatments (e.g. diets, vitamins), while beliefs that their child’s condition was cyclical in nature or attributing illness in pregnancy to being the cause of their child’s autism was associated with increased chance of using medication (Al Anbar et al., 2010; Dardennes et al., 2011). Finally, illness representations can be addressed and changed through intervention in order to promote illness adaptation and self-management: for example, psychological interventions based on CSM may help improve treatment adherence through changing the *control/cure* dimension (Jones et al., 2016).

Typically, the CSM has been applied in exploring illness perceptions in individuals with acute physical health issues and chronic conditions, including myocardial infarction (Moss-Morris et al., 2002), cancer (Richardson et al., 2017), inflammatory bowel disease (Hayes et al., 2020) and asthma (Sonney et al., 2016), and explored the illness representations of caregivers too (e.g. parents and spouses of person with chronic condition) (Law et al., 2014; Quinn et al., 2017). In recent years, the CSM has been applied to mental disorders such as schizophrenia (Lobban et al., 2004), depression and anxiety (Bear et al., 2021; Fortune et al., 2004) and neurodevelopmental disorders such as autism (Al Anbar et al., 2010; Dardennes et al., 2011) and attention-deficit hyperactivity disorder (ADHD) (Ringer, 2021; Wong et al., 2018). As neurodevelopmental conditions typically emerge during childhood, the illness perceptions of parents/caregivers are important to consider due to their ‘gatekeeper’ role in recognising a health issue and accessing healthcare and treatment for their child. While a child with a long-term condition subjectively experiences their health issue, their parent/caregiver vicariously experiences it too (Sonney et al., 2016). For chronic conditions that start in childhood, there may be ‘shared management’ between child and parent, with changes in roles and rebalancing of responsibilities plus increased autonomy for the child as they enter adolescence and adulthood (Sonney et al., 2016). To date, research has applied the CSM in exploring illness representations in people with neurodevelopmental conditions and their caregivers—particularly in exploring children’s and parents’ beliefs around ADHD (Wong et al., 2018). Exploring the health beliefs of children living with neurodevelopmental conditions—and family members that are responsible for them—can potentially help identify outcomes for treatment and gaps in knowledge and beliefs that could be subsequently addressed in psychoeducational interventions (Wong et al., 2018) and potentially other intervention options. Applying theoretical models of how individuals interpret their health—such as the CSM—onto health issues not traditionally appraised through this model can help our understanding of how people living with neurodevelopmental disorders interpret their symptom-related experiences, and how these may potentially impact on their adaptation, coping and management of living with neurological disorders across the lifespan (Bear et al., 2021).

One such neurodevelopmental disorder includes tic disorders (TDs) - such as Tourette syndrome (TS)—a long-term neurodevelopmental condition requiring adaptation and management by both the child and their parents or caregivers. TDs are characterised by tics: involuntary, quick, repetitive motor movements (motor tics) or vocalisations (vocal/phonic tics) that can be simple or complex in nature. TDs typically have their onset in childhood, are more prevalent in males, and tend to decrease in late adolescence and adulthood (Ueda & Black, 2021). TS (motor and vocal tics present for ≥1 year) and chronic motor or vocal TD (motor or vocal tics present for ≥1 year) have an estimated prevalence of 0.77% and 1.61% respectively (Knight et al., 2012). As tics are a symptom in other health conditions (e.g. autism, obsessive compulsive disorder) and look like symptoms indicative of other health issues (e.g. allergies, eye problems), tics may be misattributed by clinicians and parents, resulting in incorrect referrals (Szejko et al., 2022). Furthermore, there are several common co-occurring conditions (e.g. ADHD, anxiety) which may add further difficulties in identification and management of TDs (Mingbunjerdsuk & Zinner, 2020). Current TD models suggest tics arise from altered functioning of the cortico-striatal-thalamo-cortical brain circuit, along with impaired functioning in neurotransmitters involved in controlling movement (Ueda & Black, 2021). A common misconception is that coprolalia—vocal tics of obscene or socially-inappropriate words - are a key symptom of TS; in reality, approximately a fifth of TS patients have coprolalia (Freeman et al., 2009). The frequency, intensity and severity of tics can wax and wane over time (Buckser, 2008; Harris & Singer, 2006), and can be influenced by internal and external factors (e.g. emotions, environment) (Buckser, 2008). Many individuals report experiencing a premonitory urge prior to a tic (Quezada & Coffman, 2018); while tics are involuntary in nature, there is some ability to suppress or delay it through this premonitory urge (Buckser, 2008). Tic suppression can cause pain and discomfort, and the use of tic suppression may influence health beliefs surrounding the cause, impact and severity of tics (Mingbunjerdsuk & Zinner, 2020). TDs are not curable but symptoms can be managed through intervention: behavioural therapies involve focusing on the premonitory urge to reduce tics (Verdellen et al., 2011), while pharmacotherapy can be effective in reducing the frequency and severity of tics, but can have unfavourable side effects (Quezada & Coffman, 2018). As tics are often visible, people with TDs often find themselves the focus of unwanted scrutiny and attention from others (Buckser, 2008). TDs are often misunderstood and lead to discrimination, bullying and stigma (Malli et al., 2016). For example, teachers may mistake tics for disruptive behaviour or clumsiness, and misattribute their cause to poor parenting (Mingbunjerdsuk & Zinner, 2020).

As TDs typically emerge in childhood, parents’ health beliefs should also be considered when evaluating how theirs and the family’s perceptions could potentially impact upon recognition, management, and treatment of their child’s tics. Parents and family’s illness representations may shape their child’s health beliefs and how they perceive and understand their condition (Heyduck-Weides et al., 2019). The aim of this systematic review was to identify evidence focusing on the illness perceptions of TDs among children, young people and adults with tics/TDs, and illness perceptions of parents/caregivers to children with TDs. To the best of our knowledge, this is the first review to focus on TDs through the theoretical lens of illness representations offered by the CSM. Doing so could support understanding of illness cognitions and coping strategies, both which impact upon illness outcomes and could be used to address illness representations and illness perceptions, which may have a role in the management of TDs over time.

## Method

### Eligibility criteria

Studies were included if they were primary quantitative and qualitative research exploring perceptions and beliefs of TDs, were published in English between 1^st^ January 1970 to 31^st^ July 2023, and reported findings that could be mapped onto at least one dimension of illness representations as outlined by the CSM. Following the same method used by Wong and colleagues (Wong et al., 2018), we took a broad approach to how measurement of illness perceptions was utilised in quantitative studies. Outcome measures had to map onto ≥1 illness representation dimension—as judged by the authors—and did not solely focus on standardised measures of illness beliefs (e.g. the Illness Perceptions Questionnaire, IPQ), given that the present review was the first to attempt mapping on the CSM to TDs and illness belief measures may not have been applied to this condition. Samples eligible for inclusion included adults, children and young people (CYP) with TDs, and parents/caregivers and relatives of individuals with TDs. Systematic reviews and meta-analyses, opinion articles, letters to editors, conference abstracts and theses were excluded.

### Data sources

A systematic search of literature published between 1^st^ January 1970 and 31^st^ July 2023 was conducted in August 2023 across MedLine, Embase, PubMed, Web of Science, PsycINFO, and CINAHL databases. The search terms were developed through discussion with authors KK and CB, and through reviewing previous systematic reviews focusing on TDs and CSM respectively (Hayes et al., 2020; Hollis et al., 2016; Khan et al., 2019; Law et al., 2014; Richardson et al., 2017; Smith et al., 2015; Wong et al., 2018). Search terms reflected tic disorders, the CSM, illness perceptions and illness beliefs (see Supplementary material 1). Other sources of evidence searched included the reference lists and forward citations of relevant systematic reviews (Evans et al., 2016; Malli et al., 2016; Nussey et al., 2013; Smith et al., 2015; Suh et al., 2022).

### Study appraisal and selection

**Figure 2.**
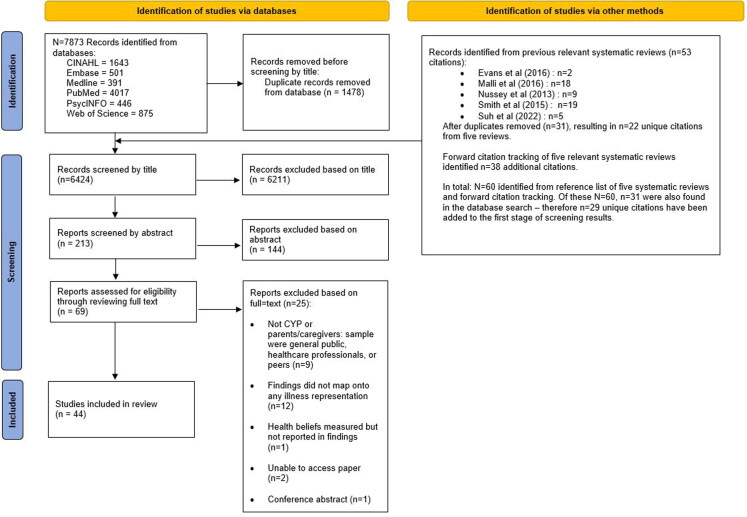
PRISMA flow diagram showing search process.

The first author (CP) carried out the initial search across the six databases and extracted results to EndNote X9. After removing duplicates, the citation titles (*N* = 6424) were screened by CP for potential eligibility following the described eligibility criteria. EBD reviewed CP’s judgments by blindly assessing a random 20% of citation titles (*n* = 1284). These were randomly selected using a random number generator (www.random.org) and found 20 titles (1.5%) had a difference in judgment between the reviewers. These differences were resolved though discussion. Next, the abstracts of citations deemed potentially eligible (*n* = 213) were reviewed separately by CP and EBD, where any disagreements were discussed and resolved. CP and EBD separately reviewed and judged the eligibility of the full texts (*n* = 69), with judgements discussed between the two reviewers in their eligibility for inclusion. Figure 2 summarises the search process.

### Data extraction

CP extracted the following data from each eligible study: citation information, study design and aim, country of study, sample information (number, age range, mean age, gender, diagnosis, type of population), data collection and analysis methods, and findings relating to one or more dimension of illness representations. Data was extracted into a Microsoft Excel document and data extraction was checked by EBD. Following a narrative synthesis of the studies, study characteristics such as the type of sample, study design, and findings relating to at least one dimension of illness representations (e.g. findings relating to TDs and at least one aspect of cognitive or emotional illness representations in the CSM) were synthesised. No statistical analyses were performed.

### Quality assessment

As the systematic review included papers that used a range of study designs, several quality appraisal checklists developed by the Joanna Briggs Institute (JBI) were used to assess study quality. Each JBI checklist presents a series of questions relating to the study’s methodological quality, which are answered with ‘Yes’, ‘No’, ‘Unsure’, and ‘Not applicable’. For qualitative research studies, the 10-item JBI critical appraisal checklist for qualitative research was used (Joanna Briggs Institute, 2020). The eight-item JBI critical appraisal checklist for analytical cross-sectional studies was used to assess cross-sectional studies (Joanna Briggs Institute, 2020). Quality assessment was performed independently by CP and KK: any discrepancies in judgements were discussed and resolved with EBD.

## Results

Forty-four studies met inclusion criteria (Supplementary Material 2). Sixteen studies recruited people with TDs: nine studies consisted of children and young people (CYP) with TDs (Cutler et al., 2009; Edwards et al., 2017; Ghanizadeh et al., 2010; Lee et al., 2016; 2019; Smith et al., 2016; Storch et al., 2007; Wadman et al., 2013; Yang et al., 2019), adults with TDs (*n* = 10) (Coleman & Melia, 2024; Conelea et al., 2013; Keiper, 1976; Lewin et al., 2012; Malli et al., 2019; Malli & Forrester-Jones, 2022; O’Connor et al., 1994; 2009; Stofleth & Parks, 2023; Taylor et al., 2022), or both (*n* = 3) (Kompoliti et al., 2006; 2009; Matsuda et al., 2016). Sixteen studies recruited parents/caregivers of CYP with TDs (Bamigbade et al., 2022; Charania et al., 2022; Claussen et al., 2018; De Lange & Olivier, 2004; Dooley et al., 1999; Espil et al., 2014; Ludlow et al., 2018; O’Hare et al., 2015; 2017; Packer, 2005; Patel et al., 2020; Pine et al., 2022; Rivera-Navarro et al., 2009; 2014; Travis & Juarez-Paz, 2020; Wolicki et al., 2019), with seven studies recruiting both CYP with TDs and parents (Cloes et al., 2017; Conelea et al., 2011; Cuenca et al., 2015; Ghanizadeh et al., 2010; Grace & Russell, 2005; Wadman et al., 2016; Zinner et al., 2012). Nineteen studies took quantitative cross-sectional designs (Charania et al., 2022; Claussen et al., 2018; Dooley et al., 1999; Cloes et al., 2017; Conelea et al., 2011; 2013 Lewin et al., 2012; Espil et al., 2014; Ghanizadeh et al., 2010; Kompoliti et al., 2006; 2009; Matsuda et al., 2016; O’Connor et al., 1994; Packer, 2005; Patel et al., 2020; Storch et al., 2007; Wolicki et al., 2019; Yang et al., 2019; Zinner et al., 2012), with 20 studies using qualitative approaches (Coleman & Melia, 2024; De Lange & Olivier, 2004; Edwards et al., 2017; Grace & Russell, 2005; Keiper, 1976; Lee et al., 2016; 2019; Ludlow et al., 2018; Malli et al., 2019; O’Connor et al., 2009; O’Hare et al., 2017; Pine et al., 2022; Rivera-Navarro et al., 2009; 2014; Smith et al., 2016; Wadman et al., 2013; Stofleth & Parks, 2023; Travis & Juarez-Paz, 2020; Bamigbade et al., 2022; Wadman et al., 2016), and five studies using mixed-methods (Cuenca et al., 2015; Cutler et al., 2009; Malli & Forrester-Jones, 2022; O’Hare et al., 2015; Taylor et al., 2022).

Table 1 outlines the findings as mapped onto the CSM illness representation dimensions. Findings relating to perceptions of *consequences* arising from TDs were the most commonly reported illness representation in studies with CYP and adults with TDs (*n* = 23) and parents/caregivers of CYP with TDs (*n* = 17). None of the included studies administered validated measures assessing illness perceptions (e.g. Illness Perceptions Questionnaire, IPQ).

**Table 1.** Overview of included studies and which illness representation dimension(s) their findings mapped onto relating to perceptions of TDs.

	Participants in included studies	
Illness representations dimension in Common Sense Model	Children, young people, and/or adults with tic disorders	Parents, caregivers and/or family members of people with tic disorders
**Identity**	Cutler et al. (2009) Edwards et al. (2017) Ghanizadeh et al. (2010) Keiper (1976) Kompoliti et al. (2006) Kompoliti et al. (2009) Lee et al. (2019) Lewin et al. (2012) Wadman et al. (2013) Wadman et al. (2016)	Cuenca et al. (2015) De Lange and Olivier (2004) Dooley et al. (1999) Ghanizadeh et al. (2010) Ludlow et al. (2018) O’Hare et al. (2015) O’Hare et al. (2017) Packer (2005) Rivera-Navarro et al. (2009) Travis and Juarez-Paz (2020)
**Cause**	Edwards et al. (2017) Lee et al. (2019)	De Lange and Olivier (2004) Wolicki et al. (2019)
**Timeline**	O’Connor et al. (2009) Wadman et al. (2013)	Packer (2005)
**Consequences**	Cloes et al. (2017) Coleman and Melia (2024) Conelea et al. (2011) Conelea et al. (2013) Cutler et al. (2009) Edwards et al. (2017) Grace and Russell (2005) Keiper (1976) Lee et al. (2016) Lee et al. (2019) Lewin et al. (2012) Malli et al. (2019) Malli and Forrester-Jones (2022) O’Connor et al. (2009) Rivera-Navarro et al. (2009) Rivera-Navarro et al. (2014) Stofleth and Parks (2023) Storch et al. (2007) Taylor et al. (2022) Wadman et al. (2013) Wadman et al. (2016) Zinner et al. (2012)	*a) Caregivers’ perceived consequences of TD on their child:* Charania et al. (2022) Claussen et al. (2018) Conelea et al. (2011) Cloes et al. (2017) De Lange and Olivier (2004) Espil et al. (2014) Grace and Russell (2005) Ludlow et al. (2018) O’Hare et al. (2015) Packer (2005) Pine et al. (2022) Rivera-Navarro et al. (2014) Wadman et al. (2016) Wolicki et al. (2019) *b) Caregivers perceived consequences on themselves/family:* Bamigbade et al. (2022) Conelea et al. (2011) Ludlow et al. (2018) O’Hare et al. (2017) Rivera-Navarro et al. (2009) Rivera-Navarro et al. (2014) Travis and Juarez-Paz (2020) Wadman et al. (2016)
**Control/cure:** **Personal control**	Coleman and Melia (2024) Conelea et al. (2013) Cutler et al. (2009) Edwards et al., 2017) Lee et al. (2016) Lee et al. (2019) Malli et al. (2019) Malli and Forrester-Jones (2022) Matsuda et al. (2016) O’Connor et al. (1994) O’Connor et al. (2009) Rivera-Navarro et al. (2014) Taylor et al. (2022) Wadman et al. (2013)	Bamigbade et al. (2022) O’Hare et al. (2015)
**Control/cure: Treatment control**	Cuenca et al. (2015) Keiper (1976) Kompoliti et al. (2006) Kompoliti et al. (2009) Lee et al. (2019) Lewin et al. (2012) Malli et al. (2019) O’Connor et al. (2009) Smith et al. (2016) Yang et al. (2019)	Cuenca et al. (2015) Patel et al. (2020)
**Emotional representations**	Coleman and Melia (2024) Conelea et al. (2011) Conelea et al. (2013) Cuenca et al. (2015) Cutler et al. (2009) Edwards et al. (2017) Grace and Russell (2005) Keiper (1976) Lee et al. (2016) Lee et al. (2019) Lewin et al. (2012) Malli et al. (2019) O’Connor et al. (1994) O’Connor et al. (2009) Rivera-Navarro et al. (2014) Stofleth and Parks (2023) Taylor et al. (2022) Wadman et al. (2013) Wadman et al. (2016	*a) Caregivers’ perceived emotional representations in their child:* Conelea et al. (2011)Grace and Russell (2005) Rivera-Navarro et al. (2014) Wadman et al. (2016) *b) Caregivers perceived emotional representations in themselves and family:* Bamigbade et al. (2022) De Lange and Olivier (2004) O’Hare et al. (2017) Ludlow et al. (2018) Rivera-Navarro et al. (2009) Travis and Juarez-Paz (2020)
**Illness coherence**	Edwards et al. (2017)	Rivera-Navarro et al. (2009) O’Hare et al. (2017) Travis and Juarez-Paz (2020)

### Identity

Nineteen studies reported findings that mapped onto the *identity* dimension of CSM in relation to TDs.

#### People living with TDs (*n* = 10)

Ten studies yielded a variety of perceived TD symptoms. CYP and adults with TDs reported motor tics as being more ‘bothersome’ than vocal tics (Ghanizadeh et al., 2010; Kompoliti et al., 2006; 2009). Adult women reported eye blinking, head jerking and throat clearing tics as being their most commonly-occurring tics, with a minority reporting coprolalia (vocal tics involving swearing and socially-inappropriate remarks) and copropraxia (motor tics involving obscene gestures) (Lewin et al., 2012). While tics were perceived as the main issue in TDs, findings from qualitative and quantitative studies found CYP with TDs also identified additional symptoms, including attention and concentration difficulties, anger and rage, hyperactivity, learning difficulties, and compulsive behaviours (Cutler et al., 2009; Ghanizadeh et al., 2010; Lee et al., 2019; Wadman et al., 2016). Edwards et al (Edwards et al., 2017) found CYP used several words to describe tics, including ‘tics’, ‘habits’ and ‘behaviours’, and these labels changed as they grew older and developed a better understanding of TDs. Similarly, adults with TDs reported their tics had been labelled as ‘bad habits’ (Keiper, 1976). CYP with TS in Wadman et al. (Wadman et al., 2013) explained how other people did not believe their TS diagnosis due to not having swearing tics (coprolalia). CYP also used several terms to describe the premonitory urge associated with tics, including ‘tingle’, ‘itch’ and ‘a weird feeling’ (Edwards et al., 2017).

#### Caregivers of child with TD (*n* = 10)

Parents’ perceptions of TDs largely aligned with identity perceptions reported by people with TD. Parents perceived motor tics to be the most common symptom of their child’s TD (Dooley et al., 1999), with parents in Packer (2005) reporting eye-blinking tics as the first symptom they observed. A minority reported their child had copropraxia (24%) and coprolalia (15%) tics (Packer, 2005). Parents in Dooley et al. (Dooley et al., 1999) rated non-tic symptoms—such as learning and attention difficulties—as being more bothersome than tics. Parents in Ghanizadeh et al. (Ghanizadeh et al., 2010) perceived motor tics as the most bothersome symptom, and were more likely to state it was bothersome than their child with TS. Parents also reported TD symptomology beyond tics - including attention difficulties, behavioural issues, compulsions, learning difficulties, hyperactivity, obsessions, impulsivity, and social withdrawal (De Lange & Olivier, 2004; Dooley et al., 1999; Ghanizadeh et al., 2010; Ludlow et al., 2018; O’Hare et al., 2015; 2017). Parents perceived their child’s rage and aggression to be a challenging TD-related symptom (Dooley et al., 1999; Ghanizadeh et al., 2010; Ludlow et al., 2018). In Ghanizadeh et al. (Ghanizadeh et al., 2010), 69.6% perceived their child’s episodic rage to be their most bothersome symptom—and were more likely to state these were bothersome than their child with TS. Approx. half of parents in Dooley et al. (Dooley et al., 1999) reported their child experienced episodic rage, with 38% perceiving this to be their child’s most challenging symptom. Parents reported some challenges in recognising TD-related symptoms, including difficulties in unpicking TD-related symptoms from typical childhood behaviours (O’Hare et al., 2017), with parents in Rivera-Navarro et al. (Rivera-Navarro et al., 2009) discounting connections between their child’s TD symptoms and diagnosis due to wanting to not believe a genetic cause for their child’s condition. Eleven parents of children with TS described how both the complex nature and media misunderstandings of TDs meant people did not see true picture of TDs, and lack of diagnostic tests meant others often struggled to accept their child’s TD diagnosis (Travis & Juarez-Paz, 2020). Likewise, in an interview-based study parents reported other people assumed their child had coprolalia due to their TS diagnosis (Ludlow et al., 2018).

### Cause

#### People with TDs (*n* = 2)

Two qualitative interview-based studies with CYP with TS reported limited findings regarding their perceptions of the cause of their tics. Thirteen CYP with TS reported little understanding of what caused tics, with several causal beliefs reported - including allergies, stress, anxiety, and brain structure (Edwards et al., 2017). Three CYP with TS in Lee et al. (Lee et al., 2019) commented on perceptions of hereditary beliefs for TDs and its potential impact on future family planning.

#### Caregivers of child with TD (*n* = 2)

In a cross-sectional survey involving 115 parents of children with TS, over a quarter (29.1%) believed tics resulted from a stressful life event, with 5.9% attributing tics to an infection, and 10.4% reported being unaware that either could be a potential cause (Wolicki et al., 2019). In a qualitative study involving mothers of children with TS, one mother described her belief that her child’s TD was heritable (De Lange & Olivier, 2004).

### Timeline

#### People living with TDs (*n* = 2)

Findings from two interview-based studies found the three adults with TDs in O’Connor et al (O’Connor et al., 2009) perceived their TD as having a chronic timeline, while six adolescents in Wadman et al. (Wadman et al., 2013) described TS as a ‘constant presence’ in their lives.

#### Caregivers of child with TD (*n* = 1)

One international survey with 69 parents of CYP with TD reported a mixed prognosis of their child’s tics: 30% reported no improvement over time, 28% reported some improvement, and 23% perceived their child’s tics as deteriorating over time (Packer, 2005).

### Consequences

Findings mapping onto the consequences dimension was the most commonly reported CSM dimension found in the included studies (*n* = 34). Five studies reported *consequences* findings from both people with TDs and their caregivers (Conelea et al., 2011; Grace & Russell, 2005; Rivera-Navarro et al., 2009; 2014; Wadman et al., 2016)

#### People living with TDs (*n* = 22)

Numerous studies with children, adolescents and adults with TDs reported a predominantly negative impact of TDs, manifesting across many different domains of daily life- and led to stigma, discrimination, prejudice and bullying. The negative impact of tics **in school and upon education** was reported in 10 studies (Cloes et al., 2017; Conelea et al., 2011; 2013; Edwards et al., 2017; Grace & Russell, 2005; Keiper, 1976; Lee et al., 2019; Malli & Forrester-Jones, 2022; Rivera-Navarro et al., 2014; Wadman et al., 2016). CYP reported their tics as distracting and affected their ability to engage in academic work, including upon their abilities to concentrate and complete homework, interfere with studying (e.g. reading, hand-writing) and exams, poorer academic productivity, and resulted in missing classes/school (Cloes et al., 2017; Conelea et al., 2011; 2013; Edwards et al., 2017; Grace & Russell, 2005; Lee et al., 2019; Rivera-Navarro et al., 2014; Wadman et al., 2016). Exams were a source of stress that could exacerbate tics (Wadman et al., 2016). In asking 85 CYP with TS to rate their tic-related impairments by severity, the top four related to impairments at school, with concentrating on work and reading out loud rated first and second respectively (Cloes et al., 2017). Twelve adolescents reported feeling less academically able compared to their peers due to having TS (Rivera-Navarro et al., 2014).CYP with TS reported that suppressing their tics in school distracted them from learning (Grace & Russell, 2005; Wadman et al., 2016). People with tics reported their teachers or schools were not understanding or accommodating of their tics, resulting in conflict with teachers (Rivera-Navarro et al., 2014), unhelpful responses from teachers (e.g. being told off, asked to stop tics) (Grace & Russell, 2005; Wadman et al., 2016), being bullied by teachers (Malli & Forrester-Jones, 2022), being punished (Keiper, 1976), and being removed from class or school (Grace & Russell, 2005; Malli & Forrester-Jones, 2022; Wadman et al., 2016). Likewise, tics caused tension with other classmates in school (Grace & Russell, 2005; Wadman et al., 2016). Some schools provided suitable accommodations (e.g. extensions), and along with supportive teachers, meant students could still excel academically (Grace & Russell, 2005; Keiper, 1976).

Another noticeable *consequenc*e was upon **employment and work**: CYP reported concerns about how their tics would impact upon their future employment and career plans (Lee et al., 2019; Wadman et al., 2013). Adults with TDs described a number of employment-related consequences, including productivity, attaining and sustaining jobs (Conelea et al., 2013; Keiper, 1976; Malli et al., 2019; Malli & Forrester-Jones, 2022), and failing or being excluded from career opportunities or advancing in their current role (Conelea et al., 2013; Keiper, 1976; Lewin et al., 2012; Malli et al., 2019; Malli and Forrester-Jones, 2022). Almost a tenth (8.9%) of adults in one cross-sectional study reported being dismissed from their job due to their tics (Conelea et al., 2013), and over half (53.8%) of adults reported not applying for jobs or educational opportunities for fear of discrimination (Malli & Forrester-Jones, 2022).

The negative consequences of TDs upon **social lives, family relationships, and friendships** were evident across all ages (Conelea et al., 2011; 2013; Keiper, 1976; Lee et al., 2016; 2019; Malli et al., 2019; Malli & Forrester-Jones, 2022; O’Connor et al., 2009; Rivera-Navarro et al., 2014; Wadman et al., 2013). Surveys of CYP and adults with TDs reported mild tic-related impairment upon their social lives, family relationships, friendships and romantic relationships (Coleman & Melia, 2024; Conelea et al., 2011), with two-thirds of adults stating their TS negatively impacted their social life and ability to make and maintain friendships (Malli & Forrester-Jones, 2022) females with TDs reported significantly greater tic-related impairment upon their social lives than males with TDs (Lewin et al., 2012). Tics caused family conflict: adults with TS reported conflict arising from family members not accepting and blaming them for having TS, with this criticism resulting in poor family communication (Malli et al., 2019); while CYP with TS reported strain with their parents due to their parents’ extreme supervision and preference for their child to control their tics in public (Rivera-Navarro et al., 2014). CYP in two studies reported concerns about consequences of TDs upon future romantic relationships and starting a family (Lee et al., 2019; Wadman et al., 2013). More positively, CYP and adults with TDs reported that having tics did not stop them from taking part in activities (Keiper, 1976) or forming and maintaining friendships providing they are treated ‘normally’, and their tics are accepted as part of them (Lee et al., 2016; 2019). CYP in Wadman et al (Wadman et al., 2013) described how making friendships became easier with age as they developed coping strategies for their TS; likewise, three adults with TS reported that they personally developed and used specific strategies to manage others’ anticipated reactions (O’Connor et al., 2009).

Seven studies highlighted how people with TDs **experienced isolation arising from their condition** reported avoiding public/social situations or withdrew from them (Coleman & Melia, 2024; Conelea et al., 2013; Lee et al., 2016; Lewin et al., 2012; Malli et al., 2019; Rivera-Navarro et al., 2009; 2014; Storch et al., 2007). Women with TS described isolation arising from experiences of rejection due to TS and not having contact with other people with TS (Coleman & Melia, 2024). Compared to males with TS, females with TS were significantly more likely to avoid social events and going out in public due to tics (Lewin et al., 2012). Using the Asher Loneliness Scale, 26% of CYP with TDs screened for ‘clinically-significant’ loneliness (Storch et al., 2007), CYP and adults reported withdrawing socially in order to avoid being bullied or potential prejudice (Malli et al., 2019; Rivera-Navarro et al., 2014). Adults in Stofleth & Parks’ (Stofleth & Parks, 2023) study particularly emphasised how their tics impacted on their communication with others (e.g. misinterpreting tics as flirting).

Tied in with these negative impacts is an overarching interweaving *consequence* reflecting **experiences of stigma, discrimination, prejudice, marginalisation and/or bullying from others** (Conelea et al., 2013; Cutler et al., 2009; Edwards et al., 2017; Grace & Russell, 2005; Lee et al., 2016; 2019; Storch et al., 2007; Lewin et al., 2012; Malli et al., 2019; Rivera-Navarro et al., 2014; Stofleth & Parks, 2023; Wadman et al., 2016; Zinner et al., 2012). The visible and loud nature of tics brought unwanted attention from others in public and private places: many reported being stared at (Coleman & Melia, 2024; Edwards et al., 2017; Leventhal & Brissette, 2012; Malli & Forrester-Jones, 2022; Stofleth & Parks, 2023; Wadman et al., 2016), being verbally harassed by others (e.g. being laughed at and teased, asked to stop ticcing) (Malli & Forrester-Jones, 2022; Stofleth & Parks, 2023; Wadman et al., 2016), and many spoke of being forcibly removed or asked to leave places due to their tics (Lewin et al., 2012; Malli & Forrester-Jones, 2022; Stofleth & Parks, 2023). CYP with TDs were bullied due to their tics (Edwards et al., 2017; Zinner et al., 2012), with CYP in Cutler et al. (Cutler et al., 2009) saying their tics made them an ‘easy target’. CYP with TDs reported being taunted and teased by their peers (Cutler et al., 2009; Edwards et al., 2017; Lee et al., 2016; Grace and Russell, 2005; Wadman et al., 2016), including peers imitating their tics (Grace & Russell, 2005; Wadman et al., 2016; Zinner et al., 2012). Adults with TS described that as their school did not address incidents of bullying, it resulted in a hostile environment for them (Malli & Forrester-Jones, 2022). Storch et al. (2007) found 27% of CYP with TDs reported clinically-significant peer victimisation scores and were significantly more likely to be victimised by peers, compared to CYP with diabetes or those without a chronic condition. Adults with TDs reported different types of discrimination due to their tics, including being treated differently (68%), and asked to leave a public place (17.3%) or school (20.4%) due to tics (Conelea et al., 2013). Furthermore, adults with TD reported a range of discriminatory actions against them, including upon their education (75.4%) and in using public transport (60.8%) (Malli & Forrester-Jones, 2022). Females with TS described feeling ‘lesser than’ by other people, and being constantly judged and scrutinised led to perceived exclusion from society’ (Coleman & Melia, 2024). In contrast, CYP with TS in one study reported not being victimised or bullied by their peers (Wadman et al., 2013), and ‘acceptance’ was one reaction reported by CYP with TDs in Edwards et al. (Edwards et al., 2017).

*Consequences* of tics upon **physical health** included pain, physical injury, and muscle aches (Conelea et al., 2013; Cutler et al., 2009; Edwards et al., 2017; Lewin et al., 2012; Taylor et al., 2022) with one adult describing their tics as making them feel ‘very, very uncomfortable a lot of the time’ (Malli et al., 2019; p.829). While females reported significantly greater impact of tics on their physical health, males were 1.78 times more likely than females to report their tics resulted in pain or physical damage (Lewin et al., 2012). When exploring the impact of TDs in adults, just over a tenth (12.7%) had sought emergency treatment either for their tics or for physical injury arising from tics (Conelea et al., 2013). Adults described how the repetitiveness of tics caused pain, which would then trigger further tics (Taylor et al., 2022). Having TD had consequences on their perceived ability to do typical daily activities, including upon leisurely activities and household tasks (Conelea et al., 2011; 2013; Lewin et al., 2012). Living with TDs had consequences upon participants’ **mental health**: over half of adults believed their TD contributed towards development of a psychiatric disorder (Conelea et al., 2013). CYP reported that having TS directly decreased their quality of life (QoL) (Cutler et al., 2009), with Malli & Forrester-Jones (Malli & Forrester-Jones, 2022) finding a negative relationship between perceived QoL and experiences of discrimination in adults with TD. Women with TS described how it affected their sense of identity and struggled with what was ‘them’ and what was ‘their TS’, resulting in some difficulty in being their ‘true self’ (Coleman & Melia, 2024). Participants also identified personal growth as a psychosocial consequence of TS: they described being more genuinely empathetic and compassionate due to their lived experiences. CYP in Wadman et al. (Wadman et al., 2013) also reported positive consequences from having TS.

#### Caregivers’ perceived consequences of TD on their child (*n* = 14)

The perceived consequences of TDs identified by parents/caregivers aligned with those reported by CYP and adults with TDs: consequences included upon their child’s school/education, social lives and relationships, everyday activities, their physical and mental health, and being subjected to discrimination and stigma - with three-quarters of parents in one study perceiving their child to be discriminated against (Conelea et al., 2011). In surveys and interview-based studies, parents/caregivers particularly highlighted the impact of their child’s TD within school and educational settings—including how tics negatively affected their child’s concentration and learning, academic difficulties, assessments, participation in class, and missing learning opportunities (Claussen et al., 2018; Conelea et al., 2011; Espil et al., 2014; Grace & Russell, 2005; Ludlow et al., 2018; Packer, 2005; Pine et al., 2022; Wadman et al., 2016). Almost half of parents reported their children avoided group activities due to tics (Conelea et al., 2011), with over another half (55.5%) of parents reporting that their child’s tics hindered their ability to do activities that their peers did (Wolicki et al., 2019). Furthermore, parents mentioned how completing homework was difficult for their child due to their tics being more intense at home (Wadman et al., 2016). Parents/caregivers were aware of negative consequences of TDs on their child’s social life, establishing and maintaining friendships, social rejection, and being victims of bullying from peers (Charania et al., 2022; Cloes et al., 2017; Grace & Russell, 2005; Ludlow et al., 2018; O’Hare et al., 2015; Wadman et al., 2016). Parents perceived their child’s tics to draw unwelcome attention, making their child more self-conscious and stressed in public (Ludlow et al., 2018; O’Hare et al., 2015), with parents in Grace & Russell (Grace & Russell, 2005) observing that their child was more likely to experience peer rejection if their tics were more overt and socially unacceptable. Parents reported this resulted in their child’s lack of friends and isolation (Grace & Russell, 2005; O’Hare et al., 2015; Rivera-Navarro et al., 2014; Wadman et al., 2016), whilst over a quarter of parents reported their child being socially excluded due to tics (Packer, 2005). Parents who rated their child’s TS as ‘severe’ were more likely to report greater impairment on functioning (Wolicki et al., 2019).

Physical injuries arising from tics was noted by parents (Conelea et al., 2011; Espil et al., 2014; Ludlow et al., 2018), with 8.3% of children requiring emergency medical attention due to their tics (Conelea et al., 2011). Over two-thirds perceived their child to experience pain and injury from their tics (Conelea et al., 2011; Espil et al., 2014). In interviews, parents reported their children’s tics put them at risk of danger and injury, whilst others reported their child’s aggression was sometimes directed towards family or peers, further exacerbating bullying (De Lange & Olivier, 2004; Grace & Russell, 2005; Ludlow et al., 2018). Some contrast between adults and their children in perceived impairment was noted in Cloes et al. (Cloes et al., 2017): for example, parents ranked ‘being teased by peers’ as the most important tic-related problem their child experienced, while CYP with TS rated this as the eighth most important (out of 37 tic-related problems). Likewise, in comparing parent-rated and CYP-rated impairments, parents rated social interference more highly compared to CYP with TDs (Conelea et al., 2011).

#### Caregivers’ perceived consequences of their child’s TD on themselves and family (*n* = 8)

Eight studies reported findings from parents/caregivers about the consequences of the child’s TD upon themselves and/or family dynamics (Bamigbade et al., 2022; Conelea et al., 2011; Ludlow et al., 2018; O’Hare et al., 2017; Rivera-Navarro et al., 2014). Parents’ scores suggested their child’s TD had a negative impact on their parents’ marriage and on their siblings (Conelea et al., 2011), with findings from a qualitative study mentioning conflict between parents due to not understanding their child’s TS, which they reported contributed towards marriage breakdown (Rivera-Navarro et al., 2014). The majority (86%) of mothers in O’Hare et al. (O’Hare et al., 2017) reported that their child, themselves, or their families had become socially isolated arising from their child’s tics; parents in Rivera-Navarro et al. (Rivera-Navarro et al., 2014) also expressed similar social isolation and found it difficult to make new friendships. The child’s TD was considered in planning trips and holidays outside the home (e.g. in less public places) (Bamigbade et al., 2022; Ludlow et al., 2018), and tics disrupted family mealtimes, resulting in parents altering mealtimes (e.g. timing) to accommodate tics (Bamigbade et al., 2022). In a qualitative study, almost all mothers (90%) reported stress arising from caregiving responsibilities and being the primary carer (O’Hare et al., 2017); parents also stated this responsibility affected their own health (Rivera-Navarro et al., 2014), and a third of parents felt that they had exacerbated or developed mental health problems due to caring for child with TS (Conelea et al., 2011). Mothers also described caregiving for child with TS as ‘a struggle’ and was the ‘new normal’ for them and their families (Travis & Juarez-Paz, 2020). Relatives reported that stigma also affected the family (Rivera-Navarro et al., 2009). Findings from two qualitative studies reported parents being concerned for their child’s future (e.g. how child will manage independently) (Rivera-Navarro et al., 2014), with almost all mothers (91%) finding it challenging to be hopeful for their child’s future (O’Hare et al., 2017). Caregiving responsibilities consequently impacted their employment: parents reported having less time available to dedicate to employment and difficulties finding childcare suitable for their child’s needs (Ludlow et al., 2018; Rivera-Navarro et al., 2014); subsequently this also impacted upon their finances (Conelea et al., 2011; Ludlow et al., 2018). For example, parents also mentioned the cost of repairing items damaged/broken by their child’s tics (Ludlow et al., 2018).

### Control/cure

### Treatment control

#### People living with TDs (*n* = 10)

CYP in Lee et al. (Lee et al., 2019) believed their tics cannot be cured with medical treatments. In exploring beliefs around outcomes of treatments for tics, two-thirds of CYP wanted treatment to decrease or stop their tics or to reduce their premonitory urge to tic (Cuenca et al., 2015). Achieving a greater sense of control over tics was also considered a valuable outcome of undertaking treatment. A proportion also reported wanting treatment to help manage their emotions, both in terms of how heightened emotions impacted upon tic expression but also how having TS made them feel.

There were mixed perceptions about medication for tics. Adults reported medication to be effective for managing tics (Malli et al., 2019) with 47% of adults perceiving benefit from taking medication, but only 24.3% reporting this benefit for last for more than one year (Lewin et al., 2012). Older CYP described how medication can be useful in conjunction with psychological therapies (Smith et al., 2016). CYP also reported mixed effectiveness from medication for tics: positive experiences included reduction in tics, feeling more in control of tics, feeling less self-conscious, and being better able to camouflage their tics as other actions—with many commenting specifically on benefits of aripipazole (Cuenca et al., 2015). Negative feedback included experiencing little or zero effects of taking medication for tics, or that any positive effects were short-lived or worsened tics. A cross-sectional study found a mixture of adherence to tic medication, with 35.8% reporting ‘low’, 23.5% ‘medium’ and 40.7% reporting ‘high’ adherence (Yang et al., 2019): however no significant associations were found between these levels of adherence and their beliefs about medication necessity and concerns about medication, as measured using the Beliefs about Medicines Questionnaire (BMQ). A number of side effects arising from tic medication were reported (Cuenca et al., 2015; Keiper, 1976; Kompoliti et al., 2006; Malli et al., 2019; Smith et al., 2015): these included perceived loss of personality, drowsiness and lethargy, changes in body image and weight gain, emotional disturbances, nightmares, and a sense of not being themselves. Three CYP who reported that the negative side effects contributed to decreased sense of control over their tics (Smith et al., 2016). CYP who had not taken tic medication also reported concerns about side effects, potentially unpleasant taste, and long-term requirement for taking medication (Cuenca et al., 2015). Experiencing side effects were cited as reasons for discontinuing medication (Cuenca et al., 2015; Kompoliti et al., 2006), and some CYP discontinued or changed medication even though they perceived improvement in their tics (Cuenca et al., 2015).

Perceptions around complementary and alternative medicines (CAMs) were also explored (Kompoliti et al., 2009; Lee et al., 2016). In a cross-sectional survey of 100 TD patients and/or parents, 64% reported using at least one type of CAM (e.g. prayer, vitamins and dietary supplements, chiropractor): the most common reasons for using these CAMs was for additional benefits to their prescribed treatments (35.9%), hope that it would cure their TS (28.1%), belief that CAM was harmless (25%) or safer than medical treatments (21.9%), and preferences for ‘natural’ therapy (21.9%) (Kompoliti et al., 2009). Just over half (56%) reported that it had led to some improvement in their tics, with 41.9% reporting no difference and 1.6% reporting a detrimental effect upon tics (Kompoliti et al., 2009).

Four studies reported beliefs around psychological and behavioural interventions for TDs (Cuenca et al., 2015; Lewin et al., 2012; O’Connor et al., 2009; Smith et al., 2016). A minority (12.1%) of adults reported receiving a form of behavioural or cognitive therapy for tics, with only a small number (4.4%) perceiving this to have benefits (Lewin et al., 2012). Three adults with TS who had undergone behavioural therapy emphasised on treatment providing better control and not on ‘cure’, and to reduce trying to control others’ perceptions of them (O’Connor et al., 2009). Eight CYP in Cuenca et al. (Cuenca et al., 2015) had undertaken behavioural interventions and described it as helpful, but reported some drawbacks—such as the lengthy treatment process and difficulties in completing therapy tasks. In the same study, fourteen CYP who had not received behavioural interventions reported similar potential drawbacks, suggesting that such interventions may not work for ‘strong’ tics. Finally, Smith et al. (Smith et al., 2016) used an interpretative phenomenological analytical approach to explore CYP’s experiences of a range of talking psychological therapies for tics—this included CBT, counselling, and behavioural interventions for TDs - and perceived these psychological interventions to help improve their sense of control of the physical and emotional aspects of tics. Other perceived outcomes of psychological therapies were adjustment and acceptance of their TS, building resilience, and making sense of the ‘self’ and their lived experience in a safe place. Within therapy, CYP placed high worth on learning strategies designed to control or adapt their tics, reporting that repeated practice resulted in such strategies becoming ‘automatic’ and increased sense of control over tics. Other cognitive and behavioural strategies practised with the therapist—such as breathing techniques and challenging thinking patterns—were also perceived by CYP as beneficial in positively changing their internal state, subsequently having a calming effect upon their tics.

#### Caregivers of child with TD (*n* = 2)

Cuenca et al. (2015) conducted an online survey study involving 295 parents: their most desired outcomes for treatment were for it to reduce their child’s tic severity and frequency, help manage negative emotions arising from tics, increase their child’s control and management of tics, and increase their child’s self-esteem, confidence, knowledge and acceptance of TD. Over half (54.7%) reported their child took medication for their tics: just under half (46%) reported their child experienced ‘moderate’ or ‘severe’ side effects, including sleepiness/tiredness and weight gain. Parents rated aripiprazole as the most helpful medication and rated lowest for reported side effects. A quarter (25.9%) of parents reported their child had received behavioural therapy for tics: perceived helpfulness scores for this treatment were higher from those parents whose child had more behavioural therapy sessions. Several parents noted that their child’s age and severity of their tics impacted upon their engagement with therapy and subsequent outcomes. For parents whose child had not had behavioural therapy, several indicated that either they or their general practitioner had limited knowledge and/or access to therapy; furthermore, a small number stated there was no need for behavioural therapy as their child’s tics were manageable. In one study exploring CAMs for their child’s tics (Patel et al., 2020), the majority (69.1%) reported using at least one CAM practice, including stress management (44.6%), herbal medicine (12.7%), homeopathy (12.7%) and meditation (9.1%). The main reason for using CAM was due to not being satisfied with effects of medication for tics (either upon outcomes or due to side effects). The majority (93%) perceived CAMs to reduce the frequency of their child’s tics, with 46% stating CAMs has more beneficial than medication. Under a fifth (17%) reported side effects from CAM (e.g. mood shifts, abdominal pain, diarrhoea). By contrast, a quarter of parents (23%) who did not use CAMs were concerned that CAMs may increase tics.

### Personal control

The *personal control* aspect of *Control/Cure* dimension refers to an individual’s perceptions and beliefs about whether the health issue and symptoms can be cured or controlled through their own actions (e.g. seeking out help, medication) (Hagger & Orbell, 2022; Law et al., 2014). For this review, we expanded this dimension onto the perceived personal ability to *control* tics: the nature of tics means that while they are involuntary in nature, there is a degree of voluntary cognitive control through being able to suppress tics (Ueda et al., 2021).

#### People living with TDs (*n* = 14)

CYP with TDs reported a mix of abilities to exert control on their tics (Cuenca et al., 2015; Edwards et al., 2017; Lee et al., 2016; 2019; Wadman et al., 2013; Matsuda et al., 2016). Some CYP with TD reported a lack of control over their tics (Cuenca et al., 2015; Edwards et al., 2017), that they were not always able to control them (Lee et al., 2016), with 14% of CYP and adults in one study reporting they could not suppress tics (Matsuda et al., 2016). The wax and wane nature and unpredictability of tics meant CYP ‘lose control over their bodies’ (Lee et al., 2019; p.283). CYP described their limited perceived control over tics as letting them ‘take over’ (Cuenca et al., 2015: p.8). Varied control abilities was also reported in adults with TDs (Coleman & Melia, 2024; Keiper, 1976; Malli et al., 2019; O’Connor et al., 1994), with some adults in O’Connor et al. (O’Connor et al., 2009) also stating that they did not wish to control their tics. Several adults described their powerlessness over TS as ‘a devil on the shoulder’ (Coleman & Melia, 2024; p.15). Some studies indicated an individual’s lack of tic control was often mistaken for difficult or poor behaviour by others (Keiper, 1976). This perceived control was also prompted by other people, as some reported family members asked them to supress their symptoms in social situations (Keiper, 1976; Lee et al., 2019; Rivera-Navarro et al., 2014). People with TDs believed others misunderstood the involuntary nature of tics and felt an expectation for them to *control* their tics, with family disputes arising when this was not possible (Cutler et al., 2009; Rivera-Navarro et al., 2014).

Suppressing tics was a frequently mentioned method for controlling tics (Coleman & Melia, 2024; Edwards et al., 2017; Malli et al., 2019; Malli & Forrester-Jones, 2022; Taylor et al., 2022; Wadman et al., 2013). CYP would use suppression to delay tics from happening, meaning they could ‘release’ them in a safe place (Edwards et al., 2017; Wadman et al., 2013). However, suppression was described as physically uncomfortable, painful, and requiring substantial mental effort and concentration (Lee et al., 2016; Malli & Forrester-Jones, 2022; Matsuda et al., 2016; Wadman et al., 2013), with participants also describing how suppression lead to a ‘rebound effect’ in subsequently increasing the intensity, severity or frequency of tics (Matsuda et al., 2016; Taylor et al., 2022). CYP and adults described other methods they used to try and control tics, including disguising them as other movements (Edwards et al., 2017; Wadman et al., 2013), ignoring the tics and using distraction (Edwards et al., 2017), avoiding situations that trigger or exacerbate their tics (Lee et al., 2016), and relaxation (O’Connor et al., 2009). A small proportion of adults with TDs reported they consumed alcohol (8.5%), used tobacco (6.8%) or illegal substances (5.7%) to decrease their tics, with smaller numbers also using these methods to decrease premonitory urges (Conelea et al., 2013); ≤1% of young people reported using these substances to decrease urges (Conelea et al., 2011). In a study involving 100 CYP and adults with TS, 58% reported they always suppressed their tics or did so daily (Matsuda et al., 2016). Furthermore, the majority could suppress tics for short periods of time - less than one minute (34%) or less than 10 min (31%) - with 22% of adults reporting they could suppress for ≥1 h, compared to 9% of CYP.

#### Caregivers’ perceptions of their child’s personal control (*n* = 2)

One interview-based study with parents reported that they perceived their child’s ability to suppress, camouflage, or manage their tics allowed them to be more accepted by their peers and form better friendships (O’Hare et al., 2015). In exploring the impact of TDs upon family mealtime experiences (Bamigbade et al., 2022), mothers interviewed reported how their child would try to suppress their tics when eating outside the home, but when they were no longer able to suppress, this would bring mealtime to an abrupt finish.

### Emotional representations

#### People living with TDs (*n* = 19)

Several studies—predominately from qualitative research designs—highlight a range of emotional responses arising from having a TD. Interviews with five adults described a mix of emotional reactions to receiving a TS diagnosis, including happiness upon realising there was treatment available, suspicion due to previously being assigned incorrect medical labels, and disappointment due to perceiving the diagnosis as confirming ‘bad habits’ (Keiper, 1976). Many studies described emotions that centred on how others made them feel, or described concerns about how they are perceived by others. A common emotional response were feelings of abnormality, being ‘weird’, and being visibly different (Coleman & Melia, 2024; Conelea et al., 2011; 2013; Edwards et al., 2017; Grace & Russell, 2005; Lee et al., 2019; Lewin et al., 2012; Rivera-Navarro et al., 2014; Wadman et al., 2013). Over two-thirds of adults with TS (68%) felt ‘different’ or abnormal’ due to tics (Conelea et al., 2013), while 87% females and 78.7% males endorsed this in Lewin et al. (Lewin et al., 2012), with females significantly more likely to report feeling abnormal/different due to tics. Just under two-thirds of CYP (62.1%) reported they felt ‘different’ or ‘abnormal’ due to tics (Conelea et al., 2011). Women with TS reported how their stigmatising experiences arising from having TS affected how they perceived their tics, viewing them as indicating ‘something inherently wrong with them’ (Coleman & Melia, 2024, p. 8). Young people with TS reported reluctance to meeting peers with TS as felt dismayed at being grouped with similar ‘rejected’ individuals (Rivera-Navarro et al., 2014). People with TD also reported feeling self-conscious and how they presented themselves to other people because of their tics (Coleman & Melia, 2024; O’Connor et al., 2009). Emotional responses felt by CYP and adults with TD included embarrassment (Coleman & Melia, 2024; Edwards et al., 2017; Malli et al., 2019), shame (Lee et al., 2016; 2019), and hopelessness (Taylor et al., 2022). Adult women described how living with TS (e.g. living with other people’s responses to their tics, being perceived as ‘attention seeking’) contributed towards personal feelings of inauthenticity, contributing towards how they made sense of themselves and their self-identity (Coleman & Melia, 2024). Adolescents with tics generally perceived parents to be more concerned about their tics because of others’ reactions, making them feel self-conscious (Rivera-Navarro et al., 2014). Findings from qualitative studies reported people felt frustrated and irritated due to their tics (Coleman & Melia, 2024; Edwards et al., 2017; Keiper, 1976; O’Connor et al., 1994; Rivera-Navarro et al., 2014), discomfort from being target of unwanted attention (Stofleth & Parks, 2023), and feelings of anxiety and worry (Coleman & Melia, 2024; Cuenca et al., 2015; Cutler et al., 2009; Edwards et al., 2017; Malli et al., 2019; O’Connor et al., 2009; Wadman et al., 2016). These feelings appeared to particularly arise during social situations: CYP and adults reported being worried about other people’s reactions to their tics, fear about how their tics might affect other people, and anxiety about tics happening during school (Cuenca et al., 2015; Edwards et al., 2017; Lee et al., 2016; O’Connor et al., 2009; Wadman et al., 2016). CYP described how the uncontrollable nature of tics and lack of bodily control led to them feeling panic (Lee et al., 2016).

In contrast, some positive emotions arising from living with TD were reported. Over a quarter of adults (28.9%) reported that they felt positively ‘unique’ or ‘special’ due to tics (Conelea et al., 2013). CYP described how having TS helped develop maturity and hardiness (Lee et al., 2019); similarly, one adult described how they perceived their TD experience as ‘empowering’ and felt more confident to express themselves (Coleman & Melia, 2024). Several diagnosed children did not feel strong negative emotions towards their TD and appeared not to mind having tics (Edwards et al., 2017).

#### Caregivers’ perceived emotional representations in their child (*n* = 4)

Caregivers reported a range of negative emotions that aligned with people with TDs. Parents perceived their child’s tics to cause their child many negative emotions, such as feelings of loneliness (Grace & Russell, 2005), embarrassment (Rivera-Navarro et al., 2014) and hopelessness (De Lange & Olivier, 2004), as well as worry about not being able to complete school work and being in social situations (Wadman et al., 2016). Almost three-quarters (74.7%) reported that their child felt ‘different’ or ‘abnormal’ due to their tics (Conelea et al., 2011).

#### Caregivers’ emotional representations in themselves and family (*n* = 6)

In interviews with 22 mothers, most (91%) felt traumatised by their child’s diagnosis, with some also feeling relief (68%), and a third (32%) feeling grief as they perceived themselves to be deprived of a ‘perfect’ child (O’Hare et al., 2017), whilst others were sad for their families’ altered future (Travis & Juarez-Paz, 2020). Other parents described feeling anxious about diagnosis due to misunderstandings and stigmatisation of TS (Rivera-Navarro et al., 2009). Contrastingly, several parents felt relieved at diagnosis, grateful to have their children, or accepted their child’s TD (De Lange & Olivier, 2004; O’Hare et al., 2017). Parents in one Spanish study described healthcare professionals making them feel guilty for seeking help for their children, and felt to be making an unnecessary fuss (Rivera-Navarro et al., 2009). Parents reported feelings of guilt arising from: perceived responsibility for the genetic development of their child’s TD (De Lange & Olivier, 2004), viewing their child’s TD as a burden on their future (Travis & Juarez-Paz, 2020), or by being overtly irritated by their child’s behaviours, but realising it was due to their TD (Ludlow et al., 2018). Mothers experienced resentment and struggled in acknowledging their child’s TS (De Lange & Olivier, 2004). Over three-quarters (78%) of mothers described difficulties ‘detangling’ or separating their child’s symptoms from other challenging behaviours typically exhibited by children (O’Hare et al., 2017). Mothers described stress reactions arising from complexity of eating outside the family home due to child’s TS (Bamigbade et al., 2022). Some parents reported feeling upset when they were unsure how to deal with and respond to their child’s tics (Ludlow et al., 2018), frustrated by their own or others’ limited comprehension of TDs (Rivera-Navarro et al., 2009; Travis & Juarez-Paz, 2020), and grief over the impact of child’s TD on their own freedom (Grace & Russell, 2005). Other interviews described parents feeling isolated (Grace & Russell, 2005; Travis & Juarez-Paz, 2020), and judged by others due to their child’s behaviour, which made them feel uncertain about their parenting (De Lange & Olivier, 2004; Travis & Juarez-Paz, 2020). Another study described that nearly all (90%) mothers of diagnosed offspring had difficulty coping with feelings of anxiety stemming from their child’s tics (O’Hare et al., 2017).

### Illness coherence

A limited number of studies reported findings that mapped onto the *Illness Coherence* dimension.

#### People living with TDs (*n* = 1)

Compared to older CYP with TS, younger CYP seemed to find it more difficult to describe what tics were, which could be as a result of being younger (e.g. not having the language to describe them, not being fully aware of symptoms) (Edwards et al., 2017).

#### Caregivers of child with TD (*n* = 3)

Mothers of CYP with TS reported making efforts to understand TS and to become better educated, but commented on this learning process being a ‘struggle’ (Travis & Juarez-Paz, 2020). Just over half (55%) of mothers stated how they were the ‘educator’ in their child’s diagnosis process due to healthcare professionals having limited TD knowledge (O’Hare et al., 2017). Finally, relatives of CYP with TS reported how diagnosis was confusing due to the medical language used by healthcare professionals and limited understanding of symptoms (Rivera-Navarro et al., 2009).

### Quality assessment

All studies were appraised using two separate JBI checklists, with two papers appraised on both checklists as their included a cross-sectional survey and qualitative data (Malli & Forrester-Jones, 2022; Taylor et al., 2022). Twenty-eight qualitative studies were appraised used the JBI qualitative research checklist (Supplementary material 3); the majority (*n* = 6) met eight, *n* = 5 met nine, and *n* = 7 met all 10 criteria, suggesting the included qualitative studies had sound study design and were trustworthy. No study met less than five criteria. Sixteen did not state the studies’ philosophical perspective and so was unable to assess the availability of the researcher’s assumptions and how harmonious they were with the methodology. Twenty cross-sectional studies were appraised using the JBI checklist for analytical cross-sectional studies (Supplementary material 4). Of the eight criteria used to evaluate each study, some were not applicable to each study depending on the research question. All studies met a minimum of half the criteria. Five studies met all criteria, with *n* = 6 meeting all but one criteria. The majority of studies (*n* = 16) reported using an objective standardised method for measurement of TD (e.g. formal diagnosis)—this may have been reported by the participant themselves or by their parents/caregivers on behalf of their child. Eight studies were judged as not providing clear study inclusion criteria.

## Discussion

Through a systematic search, findings from 44 eligible studies mapped onto CSM dimensions, identifying where there is wealth of evidence—particularly for largely negative *consequences* of TDs upon individuals and their families—and where there is more limited evidence (e.g. *Cause, Timeline*). The visible and disruptive nature of tics upon the individual and environment around them leads to negative unwanted attention and judgement from other people, subsequently impacting across many aspects of daily functioning and domains, and resulting in social isolation, being stigmatised and discriminated against. Findings mapping onto *emotional representations* also demonstrated how having TDs made individuals feel; mood and anxiety disorders are leading psychiatric co-morbidities in this population (Hirschtritt et al., 2015), and compared to the general population, people with TDs are at greater risk of attempting and dying by suicide (Fernández de la Cruz et al., 2017). Although not the focus of this review, the physical and mental strain of living with a TD alongside the chronic and fluctuating nature of TDs, appears to guide coping strategies reflecting acceptance and how it is integrated into their self-identity (Maxwell-Scott et al., 2024). Furthermore, several studies reported how people with TDs purposely avoided social situations: again, this may link as a coping strategy for managing negative consequences (e.g. unwanted attention, rude comments from other people) (Maxwell-Scott et al., 2024). These findings may present an opportunity for psychosocial interventions targeting the psychological effects of these consequences. Given the reported impact of chronic tics upon identity, sense of self and self-stigma, Coleman and Melia (Coleman & Melia, 2024) suggest third-wave psychological approaches - such as Acceptance and Commitment Therapy and Compassion-Focused Therapy—may be particularly useful for this population. Additionally, addressing patients and families’ knowledge about TDs is important as misconceptions about tics (e.g. cause, prognosis) could influence treatment preference and engagement, and providing psychoeducation about the TD aetiology may help alleviate blame that parents may be placing on themselves (Wu & McGuire, 2018). Small pilot studies have explored the effects of combining ACT with behavioural therapy (Eisenhauer et al., 2025; Franklin et al., 2011) to help nurture acceptance of premonitory tic urges within the behavioural practices, but do not appear to have focused on other aspects of living with TDs. Exploring how patients make sense of their condition at commencement of psychotherapeutic or behavioural intervention may help therapists in identifying salient health beliefs impacting on patients’ well-being, which can be addressed and explored during therapy.

In terms of the *identity* of TDs, the perception of people with TDs and their caregivers largely aligned with each other: motor and/or vocal tics were at the ‘core’ of their condition, with other non-tic symptoms—including attention difficulties, anger outbursts, and hyperactivity—also reported as symptoms of TD. The depiction of TDs in the media—in particular, portraying TS as uncontrollable swearing—can affect interpretation of tic symptoms and further stigmatise TDs (Malli & Forrester-Jones, 2022). The findings reported here highlight the complexity of TDs: they go beyond tics and can be difficult to discern given societal misunderstandings and similarity to other non-tic actions/behaviours. Our search found only four studies which reported findings aligning with the *cause* dimension of the CSM, with beliefs including stress, allergies, brain structure and genetic/hereditary causes mentioned by people with TDs and their caregivers. The limited findings here cannot inform us about individuals’ causal beliefs, and how they may influence attitudes about TDs, treatment options and tic management. We can only speculate how this might link with the other CSM dimensions and how these beliefs might influence coping and treatment decisions. For example, one mother in De Lange & Olivier (De Lange & Olivier, 2004) perceived the cause of her child’s TD to be genetic, and blamed herself for her child’s condition. In turn, this self-blame might impact upon her emotional representations and trigger feelings of guilt and guide their coping strategies. Furthermore, patients’ differing causal perspectives may also reflect the neurodevelopmental nature of TDs. In a review of eleven studies exploring adolescents’ perspectives of ADHD diagnosis, Eccleston et al. (Eccleston et al., 2019) reports how some perceive ADHD medically as an intrinsic disorder or disability—while others perceive it more so as part of their identity and an individual characteristic. With TDs, the involuntary and disruptive nature of tics also impacted on participants’ sense of self, how they define their identity (e.g. seeing it as part of or separate from self) and perceived autonomy over themselves (Coleman & Melia, 2024; Cutler et al., 2009; Maxwell-Scott et al., 2024; Wadman et al., 2013).

Only three studies reported findings mapping onto the *Timeline* CSM dimension, suggesting many people with TDs viewed it to be lifelong in nature and ever-present, with some perceived variation over time in tic expression. TDs are often considered a childhood condition, with tics typically peaking in early adolescence and decreasing into early adulthood—but full remission of tics is not common (Reagan et al., 2022). In an online survey exploring patients’ experiences of using primary care for tics, both people with tics and parents reported a sense of false hope arising from their GP telling them their tics were likely dissipate with time and with no intervention (Marino et al., 2023); the difficulty in predicting the uncertain prognosis of tics can be challenging for parents to cope with (Dooley et al., 1999). The findings from this review have implications for education for patients, their families, and healthcare professionals in increasing their coherence about TD symptomology, causes, prognosis, treatment and management. The European clinical guidelines for TS and TDs outline psychoeducation as a first step for patients regardless of symptom severity (Andrén et al., 2022), and can increase knowledge, address attitudes, clarify misconceptions about TDs and its treatment (Wu & McGuire, 2018). Although not the focus of this review, several studies did report experiences with healthcare professionals having misunderstandings and lack of TD knowledge (Cuenca et al., 2015; Ludlow et al., 2018; Malli et al., 2019; Malli & Forrester-Jones, 2022; O’Hare et al., 2017; Rivera-Navarro et al., 2009; Taylor et al., 2022; Travis & Juarez-Paz, 2020). This could potentially impact on patients’ understandings, and so there may be an opportunity for increasing healthcare professionals’ TD working knowledge through appropriate training.

Focusing on appraisals of *personal control* in TDs, there was consensus that control over tic expression was possible through conscious suppression of the premonitory urge. Use of suppression came at several costs, including discomfort, pain, and disengagement due to concentrating on suppressing. Although this review did not focus on impact of illness representations upon coping strategies (as theorised in the CSM), findings from several included papers (Buckser, 2008; Coleman & Melia, 2024; Cutler et al., 2009; Edwards et al., 2017; Lee et al., 2016; 2019; Malli et al., 2019) and from a recent meta-synthesis of qualitative studies exploring coping mechanisms in individuals with TS suggest that suppression and disguising is one way of mitigating negative consequences to cope with tics (Maxwell-Scott et al., 2024). Using suppression, masking and camouflaging to ‘hide’ tics and integrate into neurotypical social situations aligns with findings for other neurodevelopmental conditions: autistic and neurodiverse adults described how masking requires considerable emotional and physical attention, and impacts upon their self-identity and inner-selves (Miller et al., 2021; Zhuang et al., 2023), while adolescents with ADHD reported concealing their diagnosis and medication to avoid victimisation and bullying (Eccleston et al., 2019). Further exploration of how individuals with TDs cope with their condition and taking a holistic approach in seeing the whole person within the societal context– and not just focusing on tic severity, frequency and impairment (Bervoets et al., 2023) - could guide psychological interventions for this group. Finally, this review included children, young people and adults with TDs—and in appraising the included studies, there appeared to be limited exploration in older adults with TDs. TDs are typically considered a childhood condition—with tics fading or minimising into adulthood - but their neurodevelopmental basis and waxing and waning nature means tics can persist into adulthood. The perspectives, and health beliefs of older individuals with TDs has largely been ignored in current research: working with this sub-group could explore illness perceptions over time, as well as exploring their support needs.

There was considerable alignment in findings between CYP with TDs and parents/caregivers, and while seven studies did recruit both CYP with TDs and parents, they were not necessarily asked the same questions regarding their perspectives on TDs. Although children with TDs are the recipient of pharmaceutical and psychological treatments, their parents are typically the ‘gatekeeper’ in recognising and responding to health problems in their child—including seeking out help from healthcare professionals and being involved in decisions around treatment. While CYP’s and parents’ perspectives about tic medication largely aligned in one included study (Cuenca et al., 2015), something to consider here is whether there is a contrast in treatment beliefs between child and parent. One study in the present review found parents perceived their child’s vocal tics and TS-associated rage to be more bothersome than their children (Ghanizadeh et al., 2010). Given this contrast, it may be that outcomes of treatment/therapy may differ between the child patient and their parents. This could potentially also impact upon treatment decisions, uptake, and adherence. For example, in using the Illness Perceptions Questionnaire (IPQ) (Weinman et al., 1996) to explore illness perceptions in parents of autistic children, those who had greater beliefs in the seriousness of ASD were more likely to use educative treatments for their child, while greater beliefs in ASD having a cyclical timeline was significantly associated with using prescribed medication (Al Anbar et al., 2010). Behavioural interventions (e.g. Exposure with Response Prevention, Habit Reversal Therapy, Comprehensive Behavioural Intervention for Tics) for tics can involve parental participation - for example, being involved in aiding their child’s behavioural practices outside the clinic (Hollis et al., 2021), and functional interventions to manage their child’s tics and their own reactions to tics (Hollis et al., 2021; Piacentini et al., 2010). Parents will have their own responses to their child’s tics and TD diagnosis and develop their own cognitive representation of TDs—given this, parents’ beliefs about TDs and their treatments also require consideration in the management of tics.

In our review, we did not identify any quantitative studies which utilised the IPQ (or similar measures) to explore illness perceptions in people with TDs or their families—and likewise, none of the included qualitative studies explicitly used the CSM to inform their study or to organise their findings. Future research may wish to explore the utility and appropriateness of the IPQ and similar outcome measures in measuring illness perceptions in this population, and their potential links with relevant health and treatment outcomes. For instance, interviews exploring CSM dimensions in children with ADHD has suggested an additional illness belief—’uniqueness’—reflecting how they position themselves to those without ADHD (Ringer, 2021). Likewise, there may be a contrast between child and parent over how bothersome the tics are, or what outcomes they would like from treatment; for example, CYP placed importance on having treatment that reduced their premonitory urge to tic, while parents did not report this being a valuable treatment outcome (Cuenca et al., 2015). Several included studies identified that TDs are typically more than ‘just’ tics, and the consequences of TDs and social stigma upon individual’s physical and mental health suggest valuable treatment outcomes other than upon tic expression.

### Limitations

Although TDs differs from diseases and illnesses that have previously been explored using the CSM (e.g. asthma, cancer, diabetes), the CSM has provided a useful framework to explore the psychosocial impact of neurodevelopmental disorders (Wong et al., 2018)—with the present review appearing to be the first attempt at applying the CSM to people with TDs and their parents/caregivers. Few studies mapped onto the *cause*, *timeline* and *coherence* domains. The mix of qualitative and cross-sectional studies provides an array of insights and different kinds of evidence, and findings from both approaches did appear to align with each other. There was a lack of longitudinal studies on this topic, which potentially could be beneficial for assessing the development and changeability of illness perceptions of TDs over time. The majority of included studies recruited participants with TD diagnosis, but a minority did include participants who had tics but not a confirmed diagnosis (e.g. (Taylor et al., 2022)). While this is not necessarily a major drawback given reported difficulties in attaining assessment and diagnosis from healthcare professionals, it is a factor to consider in evaluating the evidence. As with the systematic review of illness perceptions in ADHD (Wong et al., 2018), we did not assess potential moderating and mediating factors influencing perceptions in people with TDs and their parents/caregivers. Additionally, a large proportion of the included research was in Western Anglophone countries—models of medicine, cultural and societal factors may have an impact upon illness representations, which could be explored in future research. For example, healthcare professionals working in Uganda suggested how cultural factors may influence parents to potentially perceive their child’s tics as having supernatural causes or being ‘punishment’ from ancestors—which in turn may affect the type of help sought out (Rodin et al., 2021). Finally, we did not assess the relationship between illness representations upon coping and health outcomes of TDs, as theorised by the CSM—meaning the direct impact of these illness beliefs is unclear, but there did appear to be crossover in some beliefs and coping mechanisms (e.g. social avoidance as a *consequence* but also as a coping strategy).

## Conclusion

The CSM provided a useful framework for exploring and mapping illness representations of TDs in children, young people and adults with TDs and their caregivers. The findings highlight how beliefs about TDs and tics—both in the person themselves and in caregivers—need consideration in the recognition, assessment, diagnosis and management of TDs. Many included studies mapped onto the *consequences* and *emotional representations CSM* dimensions - particularly highlighting negative illness perceptions—with limited exploration relating to *cause* and *coherence* CSM dimensions. The findings are applicable to development of psychoeducation packages for TD patients and their families, and potentially onto healthcare professionals—as well as considering psychological interventions to support physical and mental health consequences of living with TDs.

## Supplementary Material

Supplemental Material

## Data Availability

The data that support the findings of this study are available from the corresponding author (EBD) upon reasonable request.

## Supplementary materials

- Supplementary material 1: Search terms used in the databases for the systematic review.
- Supplementary material 2: Summary of the included studies and findings relating to Common-Sense Model.
- Supplementary material 3: Quality assessment results for the included qualitative studies.
- Supplementary material 4: Quality assessment results for the included cross-sectional studies.
- Supplementary material 5: PRISMA checklist
