# Supplementary material for: A systematic review exploring perceptions of Tourette syndrome and tic disorders using the common-sense model of illness representations
Source: Psychol Health. 2025 May 14:1–34. Online ahead of print. doi: 10.1080/08870446.2025.2502515 (PMC12080458; doi:10.1080/08870446.2025.2502515)
Supplement: Supplemental Material [file GPSH_A_2502515_SM3731.zip › rev-2024-0137-File004.docx]

**Supplementary materials**

***Supplementary material 1: Search string used in the six databases.***

*(“Tourette's syndrome” OR “Tourette syndrome” OR “Gilles de la Tourette syndrome” OR “Tourette's disorder” OR “Tourette*” OR “tic disorder” OR “tic*” OR “persistent tic disorder” OR “chronic tics” OR “chronic tic disorder” OR “transient tic disorder”) AND (“view*” OR “percept*” OR “knowledge” OR “belie*” OR “attribut*” OR “understand*” OR “expect*” OR “interpret*” OR “represent*” OR “cognit*” OR “attitud*” OR “concept*” OR “Illness belief*” OR "Illness perception*” OR “Illness cognition*” OR “Illness representation*” OR “illness identit*” OR “illness belief*” OR “Illness control” OR “Illness attitude” OR “Illness course” OR “Illness consequence” OR “disease perception*” OR “disease cognition*” OR “disease representation*” OR “disease belief*” OR “disease control” OR “disease attitude” OR “health attitude”) AND (“common sense-model” OR “CSM” OR “self-regulat*” OR “self-regulation model” OR “Leventhal*” OR “Common sense self-regulatory” OR “SRM” OR “CSM” OR “CS-SRM”) NOT tick.*
