# Supplementary material for: A systematic review exploring perceptions of Tourette syndrome and tic disorders using the common-sense model of illness representations
Source: Psychol Health. 2025 May 14:1–34. Online ahead of print. doi: 10.1080/08870446.2025.2502515 (PMC12080458; doi:10.1080/08870446.2025.2502515)
Supplement: Supplemental Material [file GPSH_A_2502515_SM3731.zip › rev-2024-0137-File005.docx]

**Supplementary materials**

***Supplementary material 2: Summary of included studies and their application onto dimensions of Common Sense Model***

| **Author (Year)** | **Sample** | **Country and recruitment** | **Study design, data collection method, analysis** | **Findings as applied to dimensions of Common-Sense Model** |
| --- | --- | --- | --- | --- |
| Bamigbade et al. (2022) | N=17 mothers of children diagnosed with TS or CTD  Children:   - Age: 3-14 yrs (M NR) - Gender: 12 males, 5 females   Mothers:   - Age: NR - Gender: 17 females | United Kingdom: recruited via online adverts shared by Tourettes Action, TourettesHero and online parent support groups | Qualitative  One-to-one in-person or online interviews  Interpretative phenomenological  analysis | *Consequences:* Mothers said their child’s tics impacted on their mealtime experiences – this reflected:   1. Functional challenges: tics impaired their child’s ability to eat and drink uninterrupted – particularly head, oral, neck, throat tics. Tics also affected ‘good table etiquette’ 2. Fragmented mealtimes: tics could be disruptive to family meal times, depending on their type and severity. Tics could also harm others. Actions were taken to reduce this disruption, e.g. waiting until tics waned, the timing of meals 3. Self-consciousness and anxiety when eating out – child did not want attention on them due to tics, which subsequently influenced decisions to eat outside home (e.g. how often, location, time of eating)   *Personal control/cure:* Mothers noted that their child would try to suppress their tics when dining outside the home – the meals would be cut short when child was no longer able to suppress tics.  *Emotional representations:* Mothers spoke of stress arising from complexity of eating outside the home. |
| Charania et al. (2022) | N=51001 children and young people  n=186 had received TS diagnosis in childhood:   - Age: 6-17 yrs (M NR) - Gender: 138 male, 48 female   n=50815 did not have TS diagnosis:   - Age: 6-17 yrs (M NR) - Gender: 25921 male, 24894 female | USA: National Survey of Children’s Health 2016- 2017 – mail-in or online survey. Nationally representative sample, randomly targeted for participation.  Study was completed by their parents/ caregivers. | Quantitative:  Cross-sectional survey  Descriptive analysis,  correlations | *Consequences:* Based on data reported by their parents, CYP who had ever been diagnosed with TS were more likely to have been victim of bullying, compared to those with no TS (56.1% vs. 21.6%; p=<.001).  CYP who had ever been diagnosed with TS were more likely to have been perpetrator of bullying, compared to those with no TS (20.7% vs. 6.0%; p.04).  Children with moderate or severe TS reported greater prevalence of being both a bullying victim and perpetrator, compared with children with mild TS (34.0% vs 4.1%; p = 0.05). |
| Claussen et al. (2018) | N=129353 children and young people  n=300 had received TS diagnosis in childhood   - Age: 6-17 yrs (M NR) - Gender: 243 males, 57 females   n=129053 did not have TS diagnosis:   - Age: 6-17 yrs (M NR) - Gender: 66864 males, 62189 females | USA: recruited via telephone National Survey of Children’s Health 2007-2008 and 2011-2012.  Study was completed by their parents/ caregivers. | Quantitative  Cross-sectional survey  Descriptive analysis, regression analyses | *Consequences:* Results found no association between the presence of TS and if parents perceived their child to care about doing well in school. Results found that parents with TS reported they were significantly more likely to be contacted about problems at school and their children had significantly increased likelihood to have an individual education plan and not complete homework. |
| Cloes et al. (2017) | N=177  n=85 children diagnosed with TS:   - Age: 9-17 yrs (M 12.0) - Gender: 71 males, 14 females   n=92 children without TS:   - Age: 9-17 yrs (M 12.0) - Gender: 44 males, 47 females | USA:  Children with TS recruited via clinic at children’s hospital. Children without TS recruited via email from hospital’s marketing service.  Study was completed by children and by their parents/ caregivers. | Quantitative  Cross-sectional survey using the Child TS Impairment Scale – child and parent versions  Spearman correlations, generalised linear model regression, descriptive statistics | *Consequences:* Children with tics and their parents both rated total impairments to be higher than healthy controls and that tic impairment was influenced by tic severity. Parents’ ratings for tic impairment generally correlated with non-tic impairment.  *Consequences:* Children rated their tics to have the greatest impairment on tests, work concentration, reading/speaking out loud and mostly rated tic impairment to be greater than non-tic impairment (across 28/37 items). Children overall ranked being teased as the 8/10 for tic problems  *Consequences:* Parents rated greater non tic impairment than their children, and rated it to be greater than tic impairment (across 31/37 items). Parents perceived teasing to be the greatest tic problem and ranked problems with concentration on work to be in the top 10, four times. |
| Coleman & Melia (2023) | N=11 adults with TS diagnosis   - Age 18-28 yrs (M 22) - Gender: 11 female | United Kingdom, but recruited worldwide: advertised on social media platforms, online support groups, and Tourettes Action  Six participants based in UK, 2 USA, and 1 each in Canada, New Zealand and Philippines | Qualitative  Semi-structured one-to-one interviews via Zoom  Thematic analysis | *Consequences:* Participants described a number of societal and psychosocial consequences arising from TS. Participants described being on receiving end of negative comments and assessments from other people, and being ‘constantly subjected to external scrutiny through being stared at and being judged. The majority saw themselves as being excluded from mainstream society, using terms like “othered”, “outsider”, and “odd one out” to describe themselves. Participants reported feeling isolated due to TS – experiences of being rejected by others and not meeting peers with TS contributed to this. Participants felt ‘lesser than’, ‘pitied for’ and ‘treated delicately’ by other people, with other people assuming that they were intellectually disabled or lacked capacity. Participants described several methods they used to ‘hide’ tics from other people, including suppression, avoiding social situations, ‘blending’/masking tics into other behaviours and explaining that their tics were ‘allergies. Several described how TS hindered ability to fit into stereotypical gender roles and how women ‘should’ be in society. TS also affected participants’ sense of identity: what was ‘themselves’ and what is ‘their TS’ – felt true self was invisible, difficult to show true ‘self’ to other people and not just be labelled as ‘person with TS’. One psychosocial consequence arising from living with TS was personal growth due to being more genuinely empathetic, compassionate and being able to better relate to other people.  *Personal control/cure:* Participants used suppression to hide their tics – this (along with comorbidities) was associated with feelings of inauthenticity and uncertainty about identity. One participant felt they had inability to control tics, and several spoke about feeling ‘under siege by TS’. Several used analogy ’a devil on the shoulder’ to explain feeling ‘at the mercy of their TS’, seeing themselves as themselves as powerless or lacking autonomy over TS.  *Emotional representations:* Participants described several emotional responses to living with TS. Participants sensed that their tics meant something was ‘wrong’ with them’, and that stigmatising experiences they had been through had guided this understanding of themselves. Participants reported being highly self-conscious due to not meeting expectations about being/acting a specific way. Participants reported feelings of embarrassment, anxiety, frustration, distress and self-consciousness due to other people’s responses to their tics. They reported feeling inauthentic about themselves – contributing to this was sense that others speculated they were ‘faking’ tics or attention-seeking’ – which contributed to feeling insecure about own identity. Participants felt reduced to being a ‘condition’ and not a ‘person, and felt internalising feelings of ‘not being good enough’ due to experiences of discrimination and media representation of TS. Participants described feelings of disconnection due to TS causing them to express behaviours/tics they did not want to happen, but also described how TS was important to their sense of self – being ‘part’ of who they were. Feelings of uneasiness arose from attempts to hide their TS. One participant described her tics as ‘empowering’ – explaining how prior to her tics becoming visibly noticeable she was quiet, but TS had boosted confidence to express self. |
| Conelea et al. (2011) | N=972  n=740 parents/ caregivers of child with TD  Children:   - Age: 4-17 yrs (M 10.6) - Gender: 597 males, 143 females - Diagnosis: 230 TS, 2 CMTD   Parents/caregivers:   - 18-76 yrs (M 41.1) - Gender: 40 males, 678 females, 22 N/A   n=232 children with TD diagnosis:   - Age: 10-17 yrs (M 12.4) - Gender: 192 males, 40 females - Diagnosis: 230 TS, 2 CMTD | USA:  recruited via a link posted and sent around to members of TS Association | Quantitative  Online cross-sectional survey using TS Impact Survey for Children and Family Impact Questionnaire  Descriptive statistics | **Findings from n=232 children with TD:**  *Consequences:* Youth perceived their TDs to socially interfere with social life (3.6%), family relationships (2.7%), friendships (3.8%) as well as private leisure (3.3%), and household tasks (2.9%). Interference across academics was also described from interference with school or the work (4.4%), difficulties studying (65.0%) and doing well in class (59.6%).  *Personal control/cure:* Youth reported using alcohol, tobacco (n=3), or illegal substances (n=3) to try to reduce adverse emotional reactions occurring because of tics (e.g. shame and feeling sad and embarrassed).  *Emotional Representations:* Most youth felt abnormal or different because of tics (62.1%).  **Findings from parents/caregivers of child with TD:**  *Consequences:* Over the previous 12 months, parents perceived their child’s TD to cause socially interference across social life (4.5%), family relationships (2.5%), friendships (3.8%) as well as private leisure (3.7%), and household tasks (2.9%).  *Consequences:* Parents reported their child experienced physical interference from their tics, causing physical damage or pain (n=428) or needing urgent medical attention or hospitalisation (n=55).  *Consequences:* Parents perceived that their children avoided situations due to their tics such as vacation (16.0%), public places (37.5%), group activities (44.5%).  *Consequences:* Parents reported moderate academic interference due to tics (4.5%). Parents perceived their child’s tics to impact the school day, from missing them (3.3%), being late to school (1.5%), or having to take unscheduled breaks (13.6%).  *Consequences:* Parents described their child to experience discrimination because of their tics, from being treated differently (75.0%), requested to leave a public place (13.5%) or a school setting (20.6%). Some parents reported businesses had discriminated against their child was or rudely treated them due to their tics (20.0%).  *Consequences:* Parent’s scores from the family impact questionnaire described that their child’s TD had a negative impact on their families’ finances, marriage, and on their siblings.  *Consequences:* The majority (59.5%) perceived their child’s tics to lead to an additional emotional disorder developing in the child, or to develop or exacerbate a parent’s emotional disorder requiring professional help.  *Emotional Representations:* Most parent’s (74.7%) perceived their child’s tics to make them feel different or abnormal. |
| Conelea et al. (2013) | N=627 adults diagnosed with CTD with onset before 18yrs   - Age: 18-77 yrs (M 35.5) - Gender: 399 males, 272 females | USA: recruited via a link posted and sent around to members of TS Association | Quantitative  Online cross-sectional survey using TS Impact Survey for Children  Descriptive statistics | *Consequences:* Adults reported that they had experienced physical interference from their tic(s) that had caused pain or physical damage to self (N = 403) or meant that they required hospitalisation or urgent medical attention (N = 86).  *Consequences:* Adults reported mild social interference due to tics across their social life (3.7%), family relationships (2.2%), friendships (3.1%), romantic relationships (2.8%), and had impacted their private leisure (3.6%) and home management (2.9%). Adults also avoided certain situations because of their tics such as vacations (13.9%), social events or entertainment activities (40.7%), public places (38.4%) and group activities (42.8%).  *Consequences:* Adults reported their tics mildly interfered with work productivity (2.7%) and co-worker relationships in the past year (2.3%). In the last year, in terms of average days, adults reported their tics resulted decreased work productivity (15.4 days), missing 0.67 days of work, being late for work due to tics (1.8 days), and taking unscheduled breaks (3.1 days). Adults also reported that their tics had meant that they had failed to pursue job advancement (n=85) or avoided job interviews (n=80).  *Consequences:* Adults described mild academic interference due to tics (3.1%). Of the 221 adult who attended school in the past 12 months, academic productivity decreased for an average of 14.2 days, an average of 1.9 were missed and were late for 1.3 classes due to tics. An average of 4.7 days due to unscheduled breaks in the school day to tic, and a few dropped out of school at some point (N=63) because of tics.  *Consequences:* Adults reported experiencing discrimination because of their tics, from being treated differently (68.0%), asked to leave a public place (17.3%) or a school setting (20.4%), or treated rudely by a business (30.8%,), landlord, neighbour, or tenant association (11.9%). Some adults reported that their tics meant that they were fired from their job (8.9%).  *Consequences:* They majority (59.2%) believed their tics directly contributed to the development of another emotional disorder.  *Personal control/cure:* When coping with consequences of tics, some adults used tobacco (4.2%), alcohol (8.9%), or illegal drugs (3.1%). To decrease tics, adults used tobacco (6.8%), alcohol (8.5%), or illegal drugs (5.7%). To decrease the premonitory urge, adults used tobacco (6.4%), alcohol (6.7%) and illegal drugs (5.1%)  *Emotional Representations:* The majority that their tics made them feel different/abnormal (68%) although some felt unique/special (28.9%). |
| Cuenca et al. (2015) | N=337  a) n=295 parents of children with diagnosed TD  Children:   - Age: 5-17 yrs (M N/A) - Gender: 234 males, 61 females   Parents/caregivers:   - Age: 27-68 yrs (M 44.0) - Gender: N/A   b) n=42 children and young people with TD diagnosis:   - Age: 10-17 yrs (M 13.4) - Gender: 32 males, 10 females | United Kingdom:  recruited via Tourette's Action electronic newsletters via link, leaflets from information events, website adverts | Mixed  methods  a) cross-sectional survey with open ended questions, analysed using content analysis  b) Telephone or face-to-face interviews, analysed using thematic analysis  Data from two separate data collection methods triangulated | **Findings from children with TD:**  *Treatment control/cure:* Youth treated their tics in several ways for tics: over half used medication (n=23), whilst others used behavioural intervention (n=8), and some used neither one (n=18). Most young people (n=29) were concerned about taking medications and their limitations, but there were mixed opinions on medications for tics- some reported tics were unchanged or worsened, whilst others perceived an improvement in tics (n=18) as well as reduced self-consciousness & increased ease of disguise in public (7 young specifically commented on aripiprazole).  *Treatment control/cure:* Despite the positive effect on tics some young stopping taking medication/changed to a different drug because of the side effects (which perceived to be drowsiness, tiredness, self-reported depression, nightmares, weight gain and a sense of not being oneself). Others believed a benefit only lasted for a limited time. Concerns about using medication for tics included perceived potential side effects, unpleasant taste, problems remembering to take the drug, the requirement to take medication for a long time.  *Treatment control/cure:* Young perceived behavioural interventions to be helpful and improved *control* and management of tics, from those who had not received it (n=30), half held positive views as they perceived it to involve learning behaviours to *control* tics which they had already found to use themselves.  *Treatment control/cure:* Youth reported wanting several aims for treatment, including treatment to decrease of stop tics and premonitory urge (perceived as causing discomfort and pain) to tic (n=28), manage (reduce negative) emotional responses associated with tics (n=19), Young people reported little or no *control* of tics (n = 21) and wanted treatment to provide a sense of *control*, to make help them feel relaxed or calmed or reduce negative emotions (e.g., self-reported anxiety) associated with tics. Youth reported emotions associated with tics to be more troublesome than tics.  *Personal control/cure:* Half of CYP sample reported little or no control over their tics, describing their tics as taking over themselves as if they were independent of person and had own will.  *Emotional Representations:* Young people reported feeling worried, anxious, or stress, particularly in social situations – and which worsened their tics.  **Findings from parents/caregivers of child with TD:**  *Treatment control/cure:* Parents who reported their children took medication for tics (54.7%) (152/278) and some (46%) reported moderate or severe side effects most often: sleepiness, tiredness or drowsiness and weight gain, whilst others reported medication was helpful**.**  *Treatment control/cure:* Parents reported a range of desired treatment outcomes: reducing the severity and number of their child’s tics was rated the most important, reducing negative emotions associated with tics like frustration, anxiety, and worries (n=64), and some felt that a treatment should help control their child’s tics (19.3%). |
| Cutler et al. (2009) | N=57 children and young people diagnosed with TS   - Age: 8-17 yrs (M 11.4) - Gender: 46 males, 11 females | United Kingdom:  recruited via TS clinic at national children’s  hospital | Mixed  Methods  Cross-sectional questionnaire and focus group  Thematic analysis  Data from both methods triangulated | *Identity:* Children and young people reported symptoms problems with attention, concentration, and angry outbursts, as symptoms of TS.  *Consequences:* Children and young people reported their TS had a directly decreased their QoL and reported physical impacts of tics to be injuries, pain from repeated movements and occasional dangerous impulses.  *Consequences:* Children and young people reported their TD made them a target for bullying, name calling, teasing which some reacted aggressively and violently to. Stressful situations were worsened by triggering more tics, one youth reported their father shouting at them to stop their tics, making them worse.  *Consequences:* Children and young people reported controlling tics was attention consuming and distracting and meant that children could not fully concentrate on the external world, causing difficulties at school along with missing time in class to leave to tic.  *Personal control/cure:*  Children and young people reported symptoms had a greater impact with increased severity, or if tics were too out of their *control* to stop. Youth reported that others didn’t understand their tics were involuntary behaviours and would be asked to stop. Youth reported the need to suppress their tics to fit in with others, avoid negative attention.  *Emotional Representations:* One young person reported they sometimes wished that they didn’t have their TD. Children and young people reported feeling anxious about other’s perceptions due to receiving negative reactions to their TS. |
| De Lange and Olivier (2004) | N=7 mothers of children with TS   - Age: 37-53 yrs (M 43.6) - Gender: 7 females   Their children:   - Age: 11-16 yrs (M 14.3) - Gender: 6 males, 1 female | South Africa:  recruited via purposeful and convenient selection support group | Qualitative  Phenomenological interviews  Tesch’s method | *Cause:* One mother described their belief that TS was hereditary.  *Consequences:* Mothers reported their child being verbally and physically aggressive to themselves and siblings.  *Emotional Representations:* Mothers described a range of emotional reactions to being a parent of a child with tics including resentment, acceptance, irritation, depression, and guilt from being the cause of their child’s disorder. Other Mothers reported they were glad they have them, whilst others reported that their child made them questioned their “parenting skills” or wanted to hit their head “on the wall” or was concerned about receiving abuse from their child in their old age. One mother reported that their child’s emotional relation to having TS was feeling hopeless and that their child “wishes he was dead”. |
| Dooley et al. (1999) | N=66 parents of children diagnosed with TS  Parents:   - Age: NR - Gender: NR   Their children:   - Age: range NR, (M 11.0) - Gender: 55 males, 11 females | Canada:  recruited via authors seeing them as patients over a 6-month period. | Quantitative  Cross-sectional questionnaire  Descriptive statistics | *Identity:* Families reported they believed the most bothersome symptom to be as learning difficulties (18%) and attention deficit problems (18%), episodic rage (36%), with motor and vocal tics being rated the least bothersome. Motor tics were rated the most prevalent and rage was reported by over half of families*.* Results found no correlation between symptoms and age, apart from learning difficulties that became more common with age. |
| Edwards et al. (2017) | N=13 children and young people diagnosed with TS   - Age: 6-17 yrs (M 10.2) - Gender: 10 males, 3 females | Canada:  recruited via outpatient psychiatry clinic in children’s hospital | Qualitative  Semi-structured interviews  Thematic analysis | *Identity:* Children reported mixed descriptions of a premonitory urge, including a “tingle” (P4, P13), “itch” (P5), “pressure” (P4, P7), “squirm” (P12), with the most prevalent description of “a weird feeling” (P10, P14, P16). All quotations from page 42.  *Cause:* Children reported a mix of causal beliefs including allergies, anxiety, stress, the brain, being present at birth or just occurring spontaneously.  *Consequences:* Children reported their tics were distracting and disruptive to themselves and others, impairing their activities. Children reported their tics caused physical pain and muscle aches as well as fatigue  Children reported tics impaired social lives, including attracting unwanted attention, bullying such as name calling and social isolation.  “They [classmates] don’t want to hang around me because I’m weird. Like I know that there are inside jokes about my Tourette’s at school and ADHD” (P4, male, age 12). Some children did not know if their peers knew about their tics because they were not commented.  *Personal control/cure:* Some children reported lack of *control* over tics (n=7) whilst other children reported a range of methods to *control* tics that could be used to *control* tics, such as ignoring, suppressing them (until they can be released) or distracting themselves (by drawing). Other children reported they their disguised tics as other movements. Some viewed managing tics to be disruptive. Children described using the methods of *control* in public because they didn’t want negative reactions to their tics, instead releasing tics when they are at home where they perceived it to be safe to release tics.  *Emotional Representations:* Children described their tics making them feel embarrassed, irritated, worried about others’ perceptions of them, nervous about their constant tic-cing. One child reported that they are unbothered by their tics.  *Illness coherence:* Compared to older children and young people, younger children appeared to find it more difficult to describe what tics were – possibly due to their age (e.g. not yet having the language to describe them, not being fully aware of symptoms). |
| Espil et al. (2014) | N=524 parents of diagnosed children with CTD  Parents:   - Age: 24-63 yrs (M 44.4) - Gender: N/A   Their children:   - Age: 10-17 yrs (M 10.5) - Gender: 436 males, 88 females - Diagnosis: 508 TS, 99 CVTD, 145 CMTD | USA: recruited via link posted and sent around to members of TS Association | Quantitative  Cross-sectional survey using TS Impact Survey  Descriptive statistics | *Consequences:* Most parents (65%) reported their child’s tics caused them pain or physical harm. Parents reported that they believed their child’s tics tics had a wide range of moderately negative impacts on their child as indicated by the average ratings on the TS impact survey across the following domains: social lives (M 4.62), relationships with family (M 2.57) and friends (M 3.95), schoolwork (M 4.58), private activities and chores (M 3.80). Across other areas, parents reported their child’s tics meat that they avoided social events or entertainment (M 2.97), group activities (M = 2.99), and public places (M 1.83). |
| Ghanizadeh et al. (2010) | N=70 child-parent dyads  n=35 children and young people diagnosed with TS   - Age: 6-18 yrs (M 11.8) - Gender: 31 males, 4 females   n=35 their parents   - Age: NR - Gender: NR | Iran:  recruited via referrals to Child and Adolescent Psychiatric Clinic | Quantitative  Cross-sectional questionnaire  Descriptive statistics | **Findings from children and young people:**  *Identity:* Results from children and young people rated what they perceived the most severe symptom present in their TD to be as follows: motor tics (39.3%) and vocal tics (20.0%), inattentiveness (10.0%), hyperactivity (5.3%), learning difficulty (22.2%) and rage (26.1%). No children with obsession reported it to be the most bothersome.  **Findings from their parents:**  *Identity:* Results from parents rated what they perceived the most severe symptom present in their child’s TD to be as follows: motor tics (71.4%) and vocal tics (35.0%), inattentiveness (5.0%), obsession (36.4), hyperactivity (15.8%), learning difficulty (22.2%) and rage (69.6%). |
| Grace and Russell (2005) | N=60  n=26 children and young people diagnosed with TS   - Age: 8-15.5 yrs (M 11.0) - Gender: 21 males, - 5 females   n=34 their parents   - Age NR - Gender: 8 males, 26 females | USA and Australia:  recruitment NR | Qualitative  Semi-structured interviews conducted separately with children and their parent(s)  Thematic analysis | **Findings from children and young people:**  *Consequences:* Many children report the impacts of their tics at school: these include being disliked, receiving teasing and impersonations, problems due to writing tics, distracted due to tic suppression, avoidance of attendance, receiving unhelpful responses from teachers like being told to stop their tics, being “too stupid” to get a job and a few being removed from school.  *Consequences:* A few children reported that teachers defended their tics to their peers (although many report this is not the case and the teacher is not helpful), they performed well in coursework but not under timed conditions and some accommodations were available (including scribes, extra time, and separate exam rooms).  *Emotional Representations:* Children reported mostly negative emotional representations such as loneliness, viewed themselves "stupid” (n=6), and feeling displaced in society. A few children reported feeling confident enough to explain the reasons behind their symptoms.  *Emotional Representations:* Two children who were schooled at home had two different emotional reactions: one did not understand the reasoning and was angry, the other thought it was easier and so was glad.  **Findings from their parents:**  *Consequences:* Parents reported a wide range of academic and social impacts of their child’s tics on their children: including their child being punished for their tics, being unable to concentrate, negative psychological impact, lack of friends and being socially rejected, being teased, and joked about (not helped by their child’s response of anger).  *Consequences:* Parents found the impacts of their child’s tics on themselves meant that they became more invested in the school reported educating the staff on their child’s disorder which many thought would help with trying to get accommodations for their child. One parent reported they gave up their job to care for her child which had a severe financial impact on their family as well as negative consequences on family relationship and parent-child relationship. Parents reported they believed other children were negatively affected and one couple described assisting in class to reduce this.  *Emotional Representations:* Parents reported that their child’s tics made them lonely. Parents reported feeling grief for loss of freedom for themselves and their child, concerned about the consequences of their child’s tics for their child (such as peer rejection) (n=5), isolated and scared about their child’s tics harming their siblings. |
| Keiper (1976) | N=5 adults with diagnosis of TS   - Age: 24-54 yrs (M NR) - Gender: 5 males | USA: recruitment NR | Qualitative  Individual recollection interviews  Critical incident technique | *Identity:* Adults reported may kinds of tics including twitches, grunts, throat-clearing, and hissing. Adults reported that their tics had often been at first, misattributed as “bad habits” (p. 1640)  *Consequences:* Adults reported negative consequences of their tics including being unable to go to places where their tics were not tolerated (the library), removal from lessons for disturbing peers, punishments from teachers, peer rejection, (although n=2 described compensating for this by doing well at schoolwork), problems getting the jobs that they were qualified for or better jobs. Adults reported concerns about finance due to the cost of treatment and future romantic relationships. Some adults reported their tics did not prevent them from having close friends and partaking in activities, but some found their peers were curious about their behaviour.  *Treatment control/cure:* Adults reported that medication in chemotherapy trials removed symptoms but replaced them with negative side effects (lack of energy and no desire to do anything) so overall did not improve their condition. They also reported concerns about becoming dependant on medication, resulting in the majority stopping it.  *Personal control/cure:* Adults reported their lack of *control* over the symptoms was sometimes seen as a lack of cooperation and described that family members asked them to *control* themselves. Adults reported several methods of managing their symptoms (which was described as exhausting) including activities like sport that improved them, disguising their tics as movements to hide them.  *Emotional Representations:* Adults reported mixed responses to their diagnosis including suspicion of it owing to previous medical experiences, elation at the prospect of treatment, calm acceptance, and disappointment as had accepted their tics as “bad habits”. Other emotions reported by adults included frustration at society, lonely and rejection (due to peers). |
| Kompoliti et al. (2006) | N=100  n=65 parent/caregiver of child with TS  n=35 adults with diagnosis of TS  Age of individuals with TS: range NR (M 19.4 yrs)  Gender: 84 males, 16 females | USA:  recruited at movement disorder centre  n=65 parents /caregivers completed study on behalf of their child. | Quantitative  Cross-sectional questionnaire  Descriptive analysis, Fisher exact test, and Wilcoxon rank sums test | *Identity:* Participants reported that the most problematic tics included motor tics (72%), vocal tics (13%), although some viewed them to be as troublesome as each other (15%)  *Treatment control/cure:* Participants reported several side effects that they experienced due to their Neuroleptic medication, most commonly general (36%), body image changes (22%), thinking/ emotion disturbances (15%), and movement disorders (14%). The most concerning side effects were risk of seizures, tardive dyskinesia, cardiac disturbances, thinking and emotional disturbances.  *Treatment control/cure:* Participants reported some stopped their medicines (n=42) for several reasons, general side effects (for 41%), movement disorders (19%), disturbances of body image (17%), thinking and emotion (17%) or gastrointestinal side effects (7%). The most common medications reported to lead to discontinuation were haloperidol (33.3%), pimozide (26.2%), and risperidone (19%).  *Treatment control/cure:* Only 3 neuroleptic side effects were accurately identified by over 75% of participants including, general symptoms (97% accuracy), body image changes (84% accuracy), movement disorders (77% accuracy).  *Treatment control/cure:* The minority met the criteria of well informed (n=47). Some patients reported they did not know any side effects (n=28), and out of these most had no exposure to neuroleptic medication (n=24). Some were well informed including those exposed to the medication (66.7%) and those not (61.2%). Participants exposed to neuroleptic treatment were better informed and less concerned about the side effects.  Some patients (45%), parents and guardians (43%) were well informed, others were not well-informed including patients (39.3%), parents and guardians (16.1%). |
| Kompoliti et al.  (2009) | N=100  n=49 parents/caregivers of child with TD   - Age: NR - Gender: NR   n=51 adults with diagnosis of TD   - Age: NR (M 21.5) - Gender: 76 males, 24 females | USA:  recruited at movement disorder centre  n=39 parents /caregivers completed study on behalf of their child.  n=24 child and parent/caregiver completed it together. | Quantitative  Cross-sectional questionnaire  Descriptive analysis | *Identity:* Participants reported the most problematic tics were motor (65%), vocal (21%) or both equally rated (14%)  *Treatment control/cure:* Participants reported to use CAM for several reasons; including reducing symptoms (39.0%) of CAM, use as an additive to already prescribed treatment (35.9%), hope for a *cure* (28.1%), believing it is harmless (25%) or safer than traditional treatments (21.9%), personal empowerment (23%), wanting a natural therapy (21.9%) or inner peace and harmony (17.2%). The type of CAM used by most participants for their TD were prayer (28), vitamins (21), and massage (19). Most patients reported CAM improved their tics (56%), but some reported CAM no change (41.9) or worsened (1.6%) their tics. Almost half reported they were happy with current treatment (46%). |
| Lee et al. (2016) | N=12 children and young people diagnosed with TS   - Age: 12-18 yrs (M 16.6) - Gender: 8 males, 4 females | Taiwan:  recruited via purposeful sampling from Taiwan Tourette Family Association | Qualitative  Individual semi-structured phenomenological interviews  Giorgi’s phenomenological methods | *Consequences:* Children and young people reported their tics led to a variety of negative consequences: including peer rejection, being mocked, and discriminated against by teachers and peers, social isolation. Children and young people reported that they care about how peers respond to their tics (so have management strategies to cope with tics) and will give up rights for their TD (e.g. military exemption) to be seen as the same as others. Children and young people reported that TS does not stop them forming friendships if they are treated normally.  *Personal control/cure:* Children and young people reported that situations are associated with onset of tics and so do their best to avoid them, these include pressure, sleep deprivation, and their emotional state. They reported that they are not always able to control tic onset and that tic suppression is very painful.  *Emotional Representations:* Children and young people reported negative emotions like they wanted to recover but they knew that their tics would keep happening and this would make them feel panicked, or they felt different to peers and so felt relieved to supress their tics. Some reported positive emotional responses arising from living with TS, such as developing self-confidence. |
| Lee et al. (2019) | N=16 children and young people diagnosed with TS   - Age: 14-20 yrs (M 17.5) - Gender: 14 males, - 2 females | Taiwan:  recruited via purposive sampling from a medical centre | Qualitative  Individual semi-structured phenomenological interviews  Giorgi’s phenomenological methods | *Cause:* Some held belief that TS is heritable, which meant some did not plan to have children.  *Consequences:* Adolescents reported negative interference of their tics on their life including conflicts with family, difficulties concentrating, writing, and reading, facing stigma, being self-conscious of their tics, and being made to have special education classes despite unaffected intelligence due to disturbing peers. Other worries include future romantic relationships, having children with TS, and future careers. Adolescents reported that provided they receive support and acceptance, their TS does not impede making friends*.*  *Treatment control/cure:* Adolescents reported they believed their tics cannot be cured despite medical treatments. One participant reported that Chinese medicine and acupuncture made no difference to their tics.  *Personal control/cure:*  One participant reported their family (father) requested their tics to be supressed.  *Emotional Representations:* Adolescents reported feeling lonely, not understood, upset, and useless. They were worried about disrupting peers learning at school, or embarrassing family, feeling sad for being different and so wanted to supress their tics. |
| Lewin et al. (2012) | N=460 adults with diagnosis of TD (TS, CMTD or CVTD) before 18 yrs old   - Age: 18-79 yrs (M 37.3 yrs in males; 34.0 in females) - Gender: 275 males, 185 females | USA: recruited via a link posted and sent around to members of TS Association | Quantitative  Cross-sectional survey using the TS Impact Survey and Yale Global Tic Severity Scale  Chi square tests and odds ratios, t-tests, descriptive analysis | *Identity:* Women reported the most common motor and vocal tics they experienced were eye blinking, head jerking, throat clearing and sniffing whilst they also reported coprolalia (10.6%) and copropraxia (6.4%).  *Consequences:* Adults declared they avoided situations due to their tics: such as public places (women= 45.8%, men= 33.0%), social events (women 1.8x more likely than men), group activities (women 1.7x more likely than men). Adults reported many tic-related negative impacts on their life including avoiding a job interview (17.7%), being treated differently (82.5%).  *Consequences:* Women reported many tic related negative impacts on their employment/job, including quitting (11.4%) not advancing (17.8%) or being fired (11.7%) from a job, being less productive (12.75 days/year), missing work (0.75 days/year), being asked to leave public spaces (18.5%), receiving rude treatment (40%). Women reported that they were more likely than men to develop depression/anxiety, or report their tics moderately interfered with private leisure activities (M = 3.8, compared to males M = 3.3), home management activities (M = 3.3) compared to than men (M =2.6).  *Treatment control/cure:* Adults reported the benefits of different treatments including medication (47%) and psychotherapy (4.4% of those who used it). Few adults reported benefit of treatment lasting over a year for medication (24.3%) and psychotherapy (1.8%).  *Emotional Representations:* Women significantly more likely to report that their tics made them feel abnormal (87%; compared to 78.8% males). |
| Ludlow et al. (2018) | N=15 parents /caregivers of child with TS   - Age NR (M NR) - Gender: 4 males, 11 females   Their children:   - Age: 7-17 yrs (M NR) - Gender: NR | United Kingdom:  recruited via advert on Tourette’s Action website | Qualitative  Semi-structured in person interviews  Thematic analysis | *Identity:* Parents reported challenging symptoms of TS to include tics, anger/rage attacks, behavioural issues. Most parents reported that others assumed their child has coprolalia when hearing about the diagnosis.  *Consequences:* Parents reported the consequences of their child’s tics - these included physical injuries, difficulties with everyday activities, lack of understanding of TS from families, and teasing about coprolalia. Parents reported academic problems due to tics such as difficulty concentrating and feeling tired from tic suppression, receiving unhelpful responses from staff, avoiding school (n=2).  *Consequences:* Parents reported the perceived impacts of child’s TS on themselves and family - these include altering holidays and leaving public spaces to avoid attention, difficulties keeping their job, finding childcare and replacing broken items (damaged due to child’s tics), leading to financial concerns.  *Emotional Representations:* Parents described many emotional responses about their child’s TS, including feelings of responsibility for other’s reactions to tics, distress when uncertain of how to cope with tics, guilt for getting angry and frustrated at tics which they misattributing to voluntary behaviour. |
| Malli et al. (2019) | N=16 adults diagnosed with TS   - Age: 24-43 yrs (M 32.6) - Gender: 12 males, 4 females | United Kingdom:  recruited via websites and social media platforms, including Tourettes Action, Tourette Focus-UK/Europe and ADHDWise UK | Qualitative  One-to-one semi-structured interviews via telephone, face-to-face, or email  Interpretative phenomenological  analysis | *Consequences:* Adults reported that their tics had a negative impact on them: such as feeling physically uncomfortable, experiencing stigma, working to higher standard to earn respect from peers and show they are as competent as people without TS, peer rejection/victimisation, bullying (name calling and humiliation) from family members, isolation from being hidden away by family or ignored by others, social withdrawal, and anxiety  *Treatment control/cure:* Adults reported medication was effective at managing their tics but most reported side effects of medication that included loss of personality and energy and feeling dreadful (mentioned haloperidol).  *Personal control/cure:* Adults described using suppression techniques to control their tics.  *Emotional Representations:* Adults reported that their TS made them feel like their life had been ruined, feeling embarrassed by the attention, shame, lonely from being misunderstood and perceiving a lack of support for adults with TS. Some adults described positive emotional responses to their TS including, undergoing personal growth, self-development and made their lives more meaningful, becoming emotionally stronger and improving them as a person. |
| Malli & Forrester-Jones (2022) | N=219 adults with diagnosis (>1 yr) of TS  a) Online survey: n=199   - Age: 18-73 yrs old, M 31.96 (SD 13) - Gender: 88 male, 92 female, 10 no answer   b) Interviews: n=20   - Age: 20-71 yrs, M 33.5 - Gender: 14 male, 6 female | United Kingdom  a) Online survey: Recruited online via short video advertisements with link to both aspects of study  b) Interviews: Online adverts (as above) and via Tourettes Action research participation registry | Mixed methods  a) Online survey, descriptive statistics, non-parametric tests  b) One-to-one interviews, thematic analysis | **Findings from online survey:**  *Consequences:* Domains which participants reported experienced most stigma and discrimination: education (75.4%), social life (71.4%), making or keeping friends (68.3%), public transport (60.8%), family (57.8%), dating (57.3%) and getting a job (54.3%). Few reported discrimination in relation to medical treatment, housing, parenting, personal security and safety, and dealing with police. Just over half (53.8%) did not apply for jobs or educational opportunities (50.3%) for fear of being discriminated against. There were no statistical differences between discrimination and demographic variables, but those with comorbidities reported more discrimination than those with no comorbid conditions (p=0.012). A negative correlation was found between perceived quality of life and discrimination.  **Findings from interviews:**  *Consequences:*  Participants described many stigmatising and discriminatory incidents in interpersonal relationships, ranging from unintentional ‘small’ acts to obvious discrimination. Participants described being stared at in public (e.g.streets, shops, restaurants) and being verbally victimised on public transport. On some occasions, participants were asked to leave public places due to others misconceiving that they were ‘under the influence’. Participants described structural stigma through discrimination they experienced in public and private institutions – many described experiences of workplace discrimination, affecting their ability to obtain and maintain employment, including being denied work development opportunities and flexible working arrangements. Barriers in education were also reported, including not being provided with reasonable accommodations/adjustments for examinations and assignments. Three participants reported being excluded from school. Most reported being bullied when at school, by their peers and their teachers – and as these incidents were not addressed, school was perceived as a hostile environment for those with TS.  *Personal control/cure:* The majority described suppressing their tics in an attempt to ‘assimilate to the norm’ and minimise their discomfort to others – suppression was described as physically and mentally exhausting. |
| Matsuda et al. (2016) | N=100 people with diagnosis of TS   - n=38 children and young people, n=62 adults - Age: 10-60 yrs (M 23.6) - Gender: 75 males, 25 females | Japan:  recruited from University of Tokyo Hospital, and advert sent to members of TS Association of Japan | Quantitative  Cross-sectional questionnaire  Descriptive statistics, Pearson’s correlation coefficients | *Personal control/cure:* Participants reported that they did not like to release tics in certain environments (84%), these included when surrounded by (lots of/unknown) people (32%), at school (18%), work (17%), in public or silent places (15%). Supressing tics here made tics worse elsewhere like at home (73%), although some did not report this rebound of tics.  *Personal control/cure:*  Participants (n=92) reported that tic suppression caused them discomfort (95%), required concentration (91%), and was tiring (83%)  *Personal control/cure:*  Participants reported varying frequency and success of tic suppression: some youth did it always/ daily (58%), or rarely supressed outstanding tics daily as reported by youth (21%) and adults (13%).  *Personal control/cure:* Participants reported supressing their tics for a short period, but some could sustain it for over an hour (18%), for less than 10 minutes (65%), for under a minute (34%), others could not sustain it (53%), and some could not supress tics (14%). |
| O'Connor et al. (1994) | N=13 adults with chronic motor tics   - Age:23-49 yrs (M NR) - Gender: 6 males, 7 females | Canada:  Recruitment NR | Qualitative  Participants kept diary for a week, recording / monitoring their tics  Repertory grid analysis, INGRID analysis | *Personal control/cure:* Eligibility criteria for the study was to have a chronic motor tic ‘over which the person has little or no control’ (p.152). Participants were asked to rate the degree of control over the tic on a periodic basis.  *Emotional Representations:* Adults reported their tics made them impatient and frustrated as they made tasks more challenging and time consuming. |
| O'Connor et al. (2009) | N=3 adults with TD   - Age: 26-55 yrs (M 36.7) - Gender: 2 males, 1 female | Canada:  recruited via health provider attached to a behavioural-  psychophysiological treatment protocol at a  specialized research centre | Qualitative  One on one interviews  Giorgi’s phenomenological reduction approach | *Timeline:*  Adults perceived their condition as chronic in nature.  *Consequences:* Adults reported that their tics led to their exclusion because they are different, and were concerned about disturbing, upsetting, or being judged or rejected by their peers.  *Treatment control/cure:*  The adults who had undergone behavioural therapy emphasised on treatment providing better control and not on ‘cure’, and to reduce trying to control others’ perceptions of them/their tics.  *Personal control/cure:* Adults reported some could not *control* tics, although some did not want to. Adults reported relaxation helped their tics and tension triggered them. Adults placed emphasis on controlling their condition, rather than curing it.  *Emotional Representations:* Adults reported wishing to not be different and fearing upsetting others due to their TD. |
| O’Hare et al. (2015) | N=194  a) 86 parents of children diagnosed with TS, and 108 parents of children without TS  Children:   - Age: 7-16 yrs – M TS group 11.44, M non-TS group 11.31 - Gender: TS group 73 male, 13 female; Non-TS group 79 male, 29 female   b) 22 mothers of children diagnosed with TS  Their children:   - Age: 7-19 yrs (M 12.0) - Gender: 20 males, 2 females | Australia:  recruited via advertisement and invitations posted and sent to members of TS Association | Mixed  Methods  a) Cross-section survey, content analysis  b) Semi-structured interviews via telephone, grounded theory | *Identity:* Parents reported aggression, impulsivity, a tendency to dominate peers, to behave bizarrely, incongruently, and social withdrawal to be symptoms of their child’s disorder.  *Consequences:* Results showed that parents of children with TS described greater frequencies of difficulties with peers, socialising and making friends as well as significantly higher rates of insecure attachment and neuroticism as a personality trait than the control group, making it harder to form friendships.  *Consequences:* Parents reported that their child’s TD meant their child had less interest in socialising for a number of reasons: these included having social anxiety (14%), facing stigma (45%) and unwanted attention, fearing being bullied (such as being rejected or teased) (18%) or viewed to be unlike peers.  *Consequences:* Parents reported that their child wanted more friendly (32%) and romantic relationships (particularly older children), but TDs meant friends were hard to sustain (23%) or spend lots of time with because of struggles of tic suppression (27%), also that their child had little interest of peers in class (32%).  *Personal control/cure:* Parents reported their children to be able to supress, disguise and manage their tics, particularly with peers or in class and this *control* helped form friendships |
| O'Hare et al. (2017) | N=22 mothers of children diagnosed with TS  Their children:   - Age: 7-19 yrs (M 12.0)   Gender: 20 males, 2 females  *NB: This is same sample as reported in second study within O’Hare et al. (2015)* | Australia:  Recruited via email invite sent to TS Association members | Qualitative  One on one semi-structured interviews  Inductive approach to detect emerging themes and a deductive approach to content analysis | *Identity:* Other related behaviours alongside tics reported by mothers included self-injurious behaviours, aggressiveness, episodic rage, impulsivity, and multiple comorbid diagnoses.  *Consequences:* Majority (91%) were primary caregiver for their child. Mothers reported their child’s tics meant that they became socially isolated (86%), particularly after diagnosis from making an environment at home for their child to release tics (73%) or had their worries about their child dismissed. Mothers reported negative impacts of their tics and from society’s lack of understanding to cause such as delayed or incorrect diagnosis, poor support from school, misattribution of blame and responsibility for symptoms and behaviours, relationship problems, and stigma. Mothers reported that they found it hard to be hopeful of their child having a ‘normal’ future (91%).  *Emotional Representations:* Mothers reported feeling a range of emotions from their child’s diagnosis such as relief (68%), finding it traumatic (91%) or at a loss of their ‘ideal’ child (95%). Mothers reported their child’s TS made it difficult to manage concerns, and anxiety (90%)  *Illness coherence:* Mothers reported they became experts on their child’s TD to advocate for them and to improve their lack of understanding. |
| Packer (2005) | N=69 parents/ caregivers of 71 children with diagnosed TD   - Age NR - Gender: 68 female, 1 male   Their children:   - Age: 6.5-18.7 yrs (M 11.1) - Gender: 62 males, 19 females | USA, Australia, Canada, Peru,  Puerto Rico, the United Kingdom:  recruited via parent-focused TS  online Usenet groups, TS related mail lists and discussion forums, the investigator’s website, TS association newsletters and local support group meetings | Quantitative  Cross-sectional survey  Descriptive statistics | *Identity:* Parents reported their child’s fist tic was mostly eye blinking. Parents reported that certain tics had been experienced but their children such as coprolalia (15%), echolalia (32%) and copropraxia (24%).  *Timeline:* Parents reported mixed opinions on how the frequency of their child’s tics altered with time, some improved reported substantial (19%) or some (28%) improvement whilst others reported the opposite (23%) or no change (30%).  *Consequences:* Parents reported their offspring’s TD had negative academic impact: these included difficulties reading (n=19) and writing (n=30), speaking in class for fear or fearing peer rejection (n=6).  Parents reported their offspring’s TD had a mild negative impact on relationships with friends (31%) although others viewed it to be moderate or severe (48%). Some parents reported accommodation plans for their child’s tics (72%), with an average of 4.47 accommodations per pupil such as: being able to exit the room to tic (n=9), extra time for work and exams (n=5), less homework along with taking exams separately from peers and preferential seating (n=4). Parents reported ignoring tics was the most common and important (n=43) accommodation for their child. Parents reported the outcomes from child’s tics were worsened from drawing attention to them, punishing them or sending them out of the room for Parents reported their child’s tics resulted from their complete or partial removal from class (n=4) into hospital, special education programs or home-schooling. |
| Patel et al. (2020) | N=110 parents/caregivers of children diagnosed with TS   - Age: 18+ - Gender: NR   Their children:   - Age: 5-18 yrs (M NR) - Gender: 84 males, 26 females | USA: recruited via identifying patients from electronic medical record for TS diagnosis | Quantitative  Cross-sectional questionnaire  Descriptive statistics | *Treatment control/cure:* Most parents reported using a CAM therapy (69.1%) most commonly stress management (44.6%), herbal medicine (18.2%), homeopathy (12.7%) and meditation (9.1%). Parents reported that CAM therapy helped more than medication (46%), reduced tics (93%), and very few reported tics remaining the same (5%) or worsening (2%). Parents reported some side effects of CAM (17%) including abdominal pain, diarrhoea, and mood changes. Some parents reported being unsatisfied with the side effects of drugs and so started CAM (56%) whilst others reported they didn’t want CAM to worsen tics or that their medication was helpful (23%). |
| Pine et al. (2022) | N=75 parents/caregivers of child with diagnosis of TS   - Age: NR - Gender: 61 female, 9 male   Their children:   - Age: 6-18 yrs - Gender: 26 male, 9 female, 5 non-binary | New Zealand: Via Tourettes New Zealand patient association mailing list, social media, and website | Qualitative  Online survey  Axial approach to coding and qualitative analysis | *Consequences*: Parents described how tics have interfered with their child’s learning at school, including tics impairing their concentration and being incompatible with the learning task. This resulted in their child missing learning opportunities. Common comorbidities also impacted upon their child’s ability to learn. |
| Rivera-Navarro et al. (2009) | N=24 – mixture of adults and young people diagnosed with TS, and relatives of person with TS  n=6 children and young people with TS  Age: 11-17 yrs (M 14.3)  Gender: 4 males, 2 females  n=6 adults with TS  Age: 20-47 yrs (M 28.1)  Gender: 4 males, 2 females  n=12 relatives of people with TS  Age: 39-64 yrs (M 47.9)  Gender: 2 male, 10 female | Spain: recruited via TS volunteer organisations/support groups in Madrid, Spain | Qualitative  Four focus groups held with different subgroups  Content analysis, Grounded theory | **Findings from people with TS:**  *Consequences:* People reported the consequences of tics depended on those around them so negative impacts were reported when they were rejected, teased, or misunderstood.  **Findings from relatives of people with TS:**  *Identity:* Parents reported that some who believed TS to be genetic, denied attributing their child’s symptoms to TS as did not want to acknowledge their genetic link.  *Consequences:* Relatives reported that the stigma arising from their child’s TS also affected the family too.  *Emotional Representations:* Parents reported being made to feel guilty by medical practitioners who said they were inflating their child’s symptoms and confused by the diagnosis from poor understanding of the identity and timeline of TS*.*  *Illness coherence:* Diagnosis often caused more confusion than clarity, due to the clinical/medical language used to describe it and categorisation of it as a ‘neuro-psychiatric’ disease, as well as poor understanding of TS. |
| Rivera-Navarro et al. (2014) | N=24 people with TS  n=12 children with diagnosis of TS   - Age: 11-18 yrs (M NR) - Gender: 8 males, 4 females   n=12 parents of child with TS   - Age: 45-64 yrs (M NR) - Gender: 2 males, 10 females | Spain:  recruited via TS volunteer organisations in Madrid, Spain | Qualitative  Four focus groups held with different subgroups  Qualitative description | **Findings from people with TS:**  *Consequences:* Children and young people reported that their TS impaired relationships and led them to avoid circumstances in case they were marginalised or mocked by others. Some reported hiding their TS.  *Personal control/cure:* Some children and young reported they did not want to hide their tics in public despite being asked by parents to do so (potentially causing family arguments).  *Emotional Representations:* Children and young reported that their TD made them feel frustrated and scared of socialising with other people with TS as this made them feel as if they belonged to a group rejected by others. Children and young reported their family was more concerned than they were about their symptoms (and their effect on others) which made them feel shameful, insecure.  **Findings from parents of people with TS:**  *Consequences:* Parents reported that their child’s tics had impacts on themselves and their family: such as others in the family realising they also had TS, family conflicts (led to family breakdown), difficulties with their occupation (as less time for it, and difficulties finding suitable childcare), social isolation making it hard to form friends, and development of health problems. Parents reported that the TD had negative impacts on their child such as social isolation, social stigma, and receiving inadequate support from schools. Parents reported concerns for their child’s future and care without them plus the development of other comorbidities.  *Emotional Representations:* Parents reported feeling concerned for their offspring’s future and that their children were embarrassed about their tic when in public. |
| Smith et al. (2016) | N=7 children and young people with tics (no specific diagnosis required for study), who had received a psychological intervention (<2 yrs) for their tics and perceived it as successful/helpful for tics  Age:   - 10-17 yrs (M NR) - Gender: 5 males, 2 females - Diagnosis: 5 TS, 1 transient TD, 1 had tics | United Kingdom:  recruited via charity Tourettes Action and 2 specialist TD clinics in London, websites, and support groups with Tourettes Action, postal distribution, via staff in clinics | Qualitative  Face-to-face semi-structured interviews  Interpretive phenomenological analysis | *Treatment control/cure:* Children and young people reported learning helpful strategies (such as using methods like mindfulness and cognitive and behavioural approaches) improved physical and emotional *control* of tics and their impacts, improve negative mood, anxiety, socialising and increase relaxation. Many reported their tics reduced.  *Treatment control/cure:* Children and young people reported practicing these strategies allowed them to overcome lack of *control* and allowed the techniques to become automatic, however some thought strategies alone were not enough and required additional therapies alongside to better *control* symptoms.  *Treatment control/cure:* Some children and young people reported medication was useful with therapy but had negative side-effects and decreased the sense of *control* of their disorder.  *Treatment control/cure:*  Children and young people reported more tic *control* was life altering allowed them to experience less disruption from their tics and stigma in public and be less isolated. Children and young people also reported increased tic *control* made the more resilient, sociable, independent, confident as well as having a more positive outlooks on themselves and their future. Youth reported that gaining more *control* of their tics allowed them to accept their occurrence instead to just wishing them to stop. |
| Stofleth & Parks (2022) | N=18 adults with TS (with and without formal diagnosis)   - Age: 19-54 yrs (M 32.4) - Gender: 8 male, 10 female | USA and Canada: recruited from an online support group for adults with TS, and snowball sampling | Qualitative  One-to-one interviews via Skype  Thematic analysis | *Consequences*: Participants described reporting six types of unwanted attention –   1. Verbal harassment – most commonly reported form of unwanted attention reported by participants, which were classified into five further categories: 2. Being laughed at due to tics 3. Being told to stop ticcing 4. Being asked if they were okay 5. Being asked what they were doing 6. Others pointing out that they were ticking 7. Being physically abused due to tics – mostly from their peers 8. Being stared at, which caused person with TS much discomfort 9. Being bullied in childhood for having TS– some experienced it in adulthood too. Perpetrators included peers, teachers, relatives, coworkers and strangers. Teachers would ridicule and mock them in front of class and embarrass them in front of peers 10. Getting into trouble – participant faced reprimand or disciplinary action from authority due to their tics 11. Being forcibly kicked out/removed from location or situation due to their tics   *Consequences*: Five participants described how their tics impacted on communication with others – tics were misinterpreted by others as romantic signals/gestures; tics caused them to pause/stop mid-sentence, meaning that the conversational partner thinks the person has stopped speaking; tics were also misinterpreted as communicative signals and felt attacked by these.  *Emotional representations*: Participants reported discomfort at being the target of unwanted attention. |
| Storch et al. (2007) | N=59 children and young people diagnosed with TS or CTD   - Age: 8-18 yrs (M 12.2) - Gender: 41 males, 18 females   This sample were compared to children with T1 diabetes and healthy controls in the same study. | USA: recruited from an OCD and tic clinic | Quantitative  Cross-sectional survey using the Schwartz Peer Victimization Scale and Asher Loneliness Scale  Descriptive analysis | *Consequences:* 27% of children and young people with TS screened for clinically-significant peer victimization by others – compared to 9% with T1 diabetes and 9% healthy controls. 26% of children and young people with TS screened for clinically-significant loneliness. |
| Taylor, Anderson & Davies (2022) | N=181 adults with diagnosed or suspected TD   - Age: 16-17yrs (M 28.4) - Gender: 58 male, 105 female, 18 non-binary | Based in United Kingdom but recruited worldwide: participants from United Kingdom, Norway, USA, Australia, Netherlands, Canada, New Zealand, Argentina, Belgium, France, Costa Rica, Finland, Germany, Guatemala, Ireland, Spain, Sweden and Uruguay  Four national TS charities shared advertisements on websites and social media; also shared to one online TS support community, and to 37 TS patient support associations | Mixed methods  Cross-sectional online survey,  Descriptive statistics, correlations, and thematic analysis of open-ended questions | *Consequences:* The most common types of pain due by tics were caused by physical effort of motor tics (97.8%), repetitive tics (77.9%) and consequences of tics (72.4%). Greatest impact of tic-related pain were upon mood, sleep, and enjoyment of life. Headaches, muscle soreness and throat soreness were frequently described tic-related pains. Bruised limbs and teeth damage were the most common injuries reported, with other tic-related consequences including arthritis, broken bones and cauliflower ear. Participants described a ‘tic-pain cycle’ – the repetitiveness of tics was main cause of pain, and this pain would then trigger further tics. Likewise, viewing/seeing injuries caused by tics could then trigger further tics.  *Personal control/cure:* Some participants discussed attempting to suppress their tics, with many stating that this made their tics more intense, more frequent or more severe – which subsequently increased pain.  *Emotional representations:* Two main patterns of psychological impact resulting from tics and pain were described – reflecting hopelessness and acceptance. Many described feeling hopelessness on their outlook in living with tic-related pain, with some describing feeling angry, experiencing unbearable pain, and some experiencing suicidal thoughts due to tic-related pain. Conversely, some participants described feeling of acceptance, which appeared to be more common in those who had less severe and/or more tolerable pain. |
| Travis and Juarez-Paz (2020) | N=11 mothers of child diagnosed with TS  Age: NR  Gender: 11 females | USA and Canada:  recruited via online support group for TS caregivers and blog post | Qualitative  Semi-structured interviews via video-conferencing  Grounded theory | *Identity*: Some mothers commented that as their child’s TS diagnosis was made from clinical observation – rather than an objective diagnostic test – this resulted in difficulties from others in accepting their child’s diagnosis.  Consequences: Mothers described caregiving for a child with TS as ‘a struggle’ and it being the ‘new normal’ for them and their families.  *Emotional Representations:* Mothers reported feeling lots of negative emotions because of their child’s disorder. This includes grief and guilt for seeing the family’s future as harder, stress and frustration from their own, their doctors and others lack understanding of TS. They also reported the need to be supported and believed by friends and family and their child’s tics left them exhausted, doubted themselves and feeling judged by others as a bad parent. Mothers reported feeling isolated due to the judgement, dismissal and poor knowledge from others and healthcare staff.  *Illness coherence:* Mothers described their efforts to understand TS better but said this learning process had been a struggle, e.g. to understand what was happening to child. |
| Wadman et al. (2013) | N=6 young people diagnosed with TS   - Age: 14-16 yrs (M NR) - Gender: 4 males, 2 females | United Kingdom:  recruited via Tourettes  Action | Qualitative  Semi-structured interviews  Interpretative phenomenological analysis | *Identity:* Some CYP reported being faced with others’ misassumptions that TS involves uncontrollable swearing, and personally knowing that TS did not mean this.  *Timeline:* CYP described TS as being a ‘constant presence’ in their lives.  *Consequences:* All children described tics having a detrimental impact on social interactions. Most worried about their future, such as side effects of medication, facing discrimination, employment struggles, and having children with TS. Some participants reported problems with friendship, but others found it easier due to developing coping strategies such as disguising tics and some reported to had good friends. One described how not having coprolalia tics meant that then people did not believe their diagnosis. None of the participants appeared to discuss peer victimisation or difficulties in meeting new individuals.  *Personal control/cure:* Young people reported an ability and need to *control* their tics through suppression, delaying them or masking them as a stretch in certain situations such as when meeting new individuals or when out in public. They reported it required lots of energy and believed that not controlling tics would cause negative consequences.  *Emotional representations*: Some CYP reported being worried about being perceived unfavourably from others due to their tics. |
| Wadman et al. (2016) | N=70 children with TS and their parents  n=35 children and young people with diagnosis of TS   - Age: 11-18 yrs (M 13.9) - Gender: 33 male, 2 female   n=35 their parents   - Age: NR - Gender: 31 female, 4 male | United Kingdom:  recruited via Tourettes  Action, and secondary schools in the East Midlands, West Midlands, and Yorkshire | Qualitative  Individual semi-structured interviews  Thematic analysis | **Findings from children and young people with TS:**  *Identity:* Youth reported anger problems were a symptom of TS.  *Consequences:* Youth reported many academic difficulties to do with their TS: these included problems with concentration when trying to supress tics (62.9 %), homework (42.8 %), exams where stress made tics worse (42.8 %), writing (25.7 %), managing anger (25.7 %) along with anxiety (28.6 %) in school, as well as unhelpful staff response to tics such as being told off or removed from class (57.1%) and difficulties with other students (57.1 %) to impersonated and made fun of them, along with being told to stop their tics. They also reported their tics can cause pain.  *Emotional Representations:* Youth reported feelings of concern, stress, and anxiety about releasing their tics in school and disrupting peers in class or exams.  **Findings from parents of children with TS:**  *Consequences:* Parents reported their child had academic difficulties due to their TS: these included problems with concentration (65.7 %), homework as when at home, fatigue worsens the tics (51.4%), exams (31.4%), writing (28.6%) managing anger causing physical harm to peers and themselves and breaking things (25.7%) along with anxiety (34.3%) in school, as well as unhelpful staff responses to tics (60.0%) and difficulties with other students (45.7 %).  *Emotional Representations:* Some parents reported being worried that their child’s tics led to social isolation, bullying in the form of physical abuse or impersonation, and having no friendships, or that their child disrupts their peers. Parents report their child is anxious about schoolwork and being in a social environments. |
| Wolicki et al. (2019) | N=115 parents/ caregivers of child diagnosed with TS   - Age: NR - Gender NR   Their children:   - Age: N/A (M 13.2) - Gender: 95 males, 20 females | USA: recruited via data from the National Survey of the Diagnosis and Treatment of ADHD and TS | Quantitative:  Cross-sectional survey  Descriptive statistics, Fisher’s exact test, t-test | *Cause:* Parents reported that they believed the cause of TS to either be a stressful life event (n=30) or an infection (e.g. strep throat; n=6) whilst some reported they did not know about the former (n=12) or latter (n=14) cause.  *Consequences:* Parents who rated their child’s TS as ‘severe’ were significantly more likely to have tics that interfered with their functioning (p=<.05), when compared to children who were rated as having ‘mild’ or ‘moderate’ TS by their parents. |
| Yang et al. (2019) | N=204 children and young people diagnosed with TD   - Age: ≤18yrs (M 7.7) - Gender: 158 males, 46 females | China: recruited via paediatric and neurology clinic | Quantitative  Cross-sectional questionnaire, administered Beliefs about Medication Questionnaire (BMQ)  Descriptive statistics | *Treatment control/cure:* Children reported that their medication adherence varied across ‘high’ (40.7%), ‘medium’ (23.5%) and ‘low’ (35.8%) adherence. There were no significant associations between adherence levels and their beliefs about medication necessity and concerns about medication, as measured using the BMQ (p=>.05). |
| Zinner et al. (2012) | N=211 parent-child dyads. Sample were children and young people with TS diagnosis and their parents / caregivers   - Age: 10-17 yrs - Gender: 169 males, 37 females   Sample were divided into two groups reflecting whether they were victims or non-victims of peer victimisation.  n=55 victims:   - Age: NR (M 12.2) - Gender: 40 males, 15 females   n=151 non-Victims:   - Age: NR (M 12.4) - Gender: 129 males, 22 females | USA:recruited via link posted on national website, emailed link to Tourette Syndrome Association members and newsletter advert  Parents/caregivers completed study on behalf of child if child aged ≤17 yrs. | Quantitative  Cross-sectional survey, descriptive statistics | *Consequences:* Results showed that a quarter (26%) of children were classified as “victims” (n=55). Victims reported that the following experiences were due to their tics: Bullying in the form of name calling, mimicking, picked on or joked about (88.6%), physical harm, starting fights (71.7%), being the subject of gossip (63%), and being deliberately excluded from activities (71.6%) |
