# Supplementary material for: A systematic review exploring perceptions of Tourette syndrome and tic disorders using the common-sense model of illness representations
Source: Psychol Health. 2025 May 14:1–34. Online ahead of print. doi: 10.1080/08870446.2025.2502515 (PMC12080458; doi:10.1080/08870446.2025.2502515)
Supplement: Supplemental Material [file GPSH_A_2502515_SM3731.zip › rev-2024-0137-File006.docx]

**Supplementary materials**

***Supplementary material 3: Quality appraisal of included qualitative studies using the JBI qualitative research checklist***

| **Reference** | **Is there congruity between the stated philosophical perspective and the research methodology?** | **Is there congruity between the research methodology and the research question or objectives?** | **Is there congruity between the research methodology and the methods used to collect data?** | **Is there congruity between the research methodology and the representation and analysis of data?** | **Is there congruity between the research methodology and the interpretation of results?** | **Is there a statement locating the researcher culturally or theoretically?** | **Is the influence of the researcher on the research, and vice- versa, addressed?** | **Are participants, and their voices, adequately represented?** | **Is the research ethical according to current criteria or, for recent studies, and is there evidence of ethical approval by an appropriate body?** | **Do the conclusions drawn in the research report flow from the analysis, or interpretation, of the data?** | **Score** |
| --- | --- | --- | --- | --- | --- | --- | --- | --- | --- | --- | --- |
| Bamigbade et al. (2022) | Yes | Yes | Yes | Yes | Yes | No | No | Yes | Yes | Yes | 8/10 |
| Coleman & Melia (2023) | Yes | Yes | Yes | Yes | Yes | Yes | Yes | Yes | Yes | Yes | 10/10 |
| Cuenca et al. (2015) | Not stated | Yes | Yes | Yes | Yes | Yes | Yes | Yes | Yes | Yes | 9/10 |
| Cutler et al. (2009) | Yes | Yes | Yes | Yes | Yes | No | No | Yes | Yes | Yes | 8/10 |
| De Lange & Oliver (2004) | Yes | Yes | Yes | Yes | Yes | No | No | Unclear | Ethical yes but does not state who provided approval | Yes | 6.5/10 |
| Edwards et al. (2017) | Not stated | Yes | Yes | Yes | Yes | No | Yes | Yes | Yes | Yes | 8/10 |
| Grace & Russell (2005) | Yes | Yes | Yes | Yes | Yes | No | No | Yes | Unclear – no mention of who provided approval | Yes | 7/10 |
| Keiper (1976) | Not stated | Yes | Yes | Yes | Yes | No | No | Yes | Not stated | Yes | 6/10 |
| Lee et al. (2016) | Yes | Yes | Yes | Yes | Yes | Yes | Yes | Yes | Yes | Yes | 10/10 |
| Lee et al. (2019) | Yes | Yes | Yes | Yes | Yes | No | Yes | Yes | Yes | Yes | 9/10 |
| Ludlow et al. (2018) | Not stated | Yes | Yes | Yes | Yes | No | No/Yes | Yes | Yes | Yes | 7.5/10 |
| Malli et al. (2019) | Yes | Yes | Yes | Yes | Yes | Yes | Yes | Yes | Yes | Yes | 10/10 |
| Malli & Forrester-Jones (2022) (nb: part of study is interviews) | Yes | Yes | Yes | Yes | Yes | Yes | Yes | Yes | Yes | Yes | 10/10 |
| O'Connor et al. (1994) | Not stated | Yes | Yes | Yes | Yes | No | No | N/A | No evidence of ethical approval | Yes | 5/10 |
| O'Connor et al. (2009) | Yes | Yes | Yes | Yes | Yes | Yes | No | Yes | Yes | Yes | 9/10 |
| O’Hare et al. (2015) | Yes | Yes | Yes | Yes | Yes | No | Yes | Unclear | Yes | Yes | 8/10 |
| O’Hare et al. (2017) | Yes | Yes | Yes | Yes | Yes | No | Yes | Yes | Yes | Yes | 9/10 |
| Pine et al. (2022) | Not stated | Yes | Yes | Yes | Yes | No | No | Yes | Yes | Yes | 7/10 |
| Rivera-Navarro et al. (2009) | Yes | Yes | Yes | Yes | Yes | No | No/Yes | Yes | Yes | Yes | 8.5/10 |
| Rivera-Navarro et al. (2014) | Yes | Yes | Yes | Yes | Yes | No | No/Yes | Yes | Yes | Yes | 8.5/10 |
| Smith et al. (2016) | Yes | Yes | Yes | Yes | Yes | Yes | Yes | Yes | Yes | Yes | 10/10 |
| Stofleth & Parks (2022) | Yes | Yes | Yes | Yes | Yes | Yes | Yes | Yes | Yes | Yes | 10/10 |
| Taylor, Anderson & Davies (2022) | Not stated | Yes | Yes | Yes | Yes | No | No | Yes | Yes | Yes | 7/10 |
| Travis & Juarez-Paz (2020) | Not stated | Yes | Yes | Yes | Yes | No | No/Yes | Unclear | Yes | Yes | 6.5/10 |
| Wadman et al. (2013) | Yes | Yes | Yes | Yes | Yes | Yes | Yes | Yes | Yes | Yes | 10/10 |
| Wadman et al. (2016) | Yes | Yes | Yes | Yes | Yes | Yes | No | Yes | Yes | Yes | 9/10 |

NB: Responses of ‘Yes/No’= Yes of the researcher on the research but not of the research on the researcher; ‘No/Yes’
