# Supplementary material for: A systematic review exploring perceptions of Tourette syndrome and tic disorders using the common-sense model of illness representations
Source: Psychol Health. 2025 May 14:1–34. Online ahead of print. doi: 10.1080/08870446.2025.2502515 (PMC12080458; doi:10.1080/08870446.2025.2502515)
Supplement: Supplemental Material [file GPSH_A_2502515_SM3731.zip › rev-2024-0137-File007.docx]

**Supplementary materials**

***Supplementary material 4: Quality appraisal of included cross-sectional studies using the cross-sectional studies checklist***

| **Reference** | **Were the criteria for inclusion in the sample clearly defined?** | **Were the study subjects and the setting described in detail?** | **Was the exposure measured in a valid and reliable way?** | **Were objective, standard criteria used for measurement of the condition?** | **Were confounding factors identified?** | **Were strategies to deal with confounding factors stated?** | **Were the outcomes measured in a valid and reliable way?** | **Was appropriate statistical analysis used?** | **Score** |
| --- | --- | --- | --- | --- | --- | --- | --- | --- | --- |
| Charania et al. (2022) | No | Yes | N/A | No | Yes – comorbidities | Yes | No | Yes | 4/7 |
| Claussen et al. (2018) | No | Yes | N/A | Yes | N/A | N/A | Unclear | Yes | 3/5 |
| Cloes et al. (2016) | Yes | Yes | Yes | Yes – DSM | Yes – comorbidities | Yes – stratification | Yes | Yes | 8/8 |
| Conelea et al. (2011) | Yes | Yes | N/A | Yes – formal diagnosis | Yes – comorbidities | Yes – split data | Yes – used established measures | Yes | 7/7 |
| Conelea et al. (2013) | Yes somewhat | Yes | N/A | Yes – formal diagnosis | No | N/A | Yes | Yes | 5/6 |
| Dooley et al. (1999) | No | Yes somewhat | N/A | Yes – DSM | No | N/A | Unclear | Yes | 3/6 |
| Espil et al. (2014) | Yes | Yes | N/A | Yes – diagnosis | No | N/A | Yes | Yes | 5/6 |
| Ghanizadeh et al. (2010) | No | Yes somewhat | N/A | Yes | No | N/A | Yes – Cronbachs alpha | Yes | 4/6 |
| Kompolti et al. (2006) | No | Yes | N/A | Yes | Yes – gender etc. | Yes – split groups | Unclear | Yes | 5/7 |
| Kompolti et al. (2009) | No | Yes | N/A | Yes | No | N/A | Yes | Yes | 4/6 |
| Lewin et al. (2012) | Yes somewhat | Yes | N/A | Yes | Unclear | N/A | Yes – valid measures | Yes | 5/6 |
| Malli & Forrester-Jones (2022) (nb: part of study was online survey) | Yes somewhat | Yes | N/A | Yes formal diagnosis | No | N/A | Yes | Yes | 5/6 |
| Matsuda et al. (2016) | Yes | Yes | N/A | Yes | Yes – age | Yes | Yes | Yes | 7/7 |
| Packer (2005) | No | Yes but not in detail | N/A | Unclear | N/A | N/A | N/A | Yes | 2/4 |
| Patel et al. (2020) | Yes | Yes | N/A | Yes – EMR | Yes – age | Yes – split groups | N/A | Yes | 6/6 |
| Storch et al. (2007) | No | Yes | N/A | Yes | Yes – gender | Yes – controlled for | Yes | Yes | 7/8 |
| Taylor, Anderson & Davies (2022) | Yes | Yes | N/A | No – self report | No | N/A | Yes | Yes | 4/6 |
| Wolicki et al. (2019) | Yes | Yes | N/A | No – self report | N/A | N/A | Unclear | Yes | 3/5 |
| Yang et al. (2019) | Yes | Yes | N/A | Yes | No | N/A | Yes | Yes | 5/6 |
| Zinner et al. (2012) | Yes | Yes | N/A | Yes – formal diagnosis | Yes – tic severity | Yes – controlled for | Yes | Yes | 7/7 |
